# A thyroid hormone regulated asymmetric responsive centre is correlated with eye migration during flatfish metamorphosis

**DOI:** 10.1038/s41598-018-29957-8

**Published:** 2018-08-16

**Authors:** Marco A. Campinho, Nádia Silva, Gabriel G. Martins, Liliana Anjos, Claudia Florindo, Javier Roman-Padilla, Ana Garcia-Cegarra, Bruno Louro, Manuel Manchado, Deborah M. Power

**Affiliations:** 1 Comparative Endocrinology and Integrative Biology Group, CCMAR, Universidade do Algarve, Campus de Gambelas, 8005-139 Faro, Portugal; 2 Instituto Gulbenkian de Ciência, Rua da Quinta Grande, 6, 2780-156 Oeiras, Portugal; 3 CBMR, Centre for Biomedical Research, Departamento de Ciências Biomedicas e Medicina, Universidade do Algarve, Campus de Gambelas, 8005-139 Faro, Portugal; 4 IFAPA Centro El Toruño, 11500 El Puerto de Santa Maria, Cádiz, Spain; 5 Present Address: IFAPA Centro El Toruño, 11500 El Puerto de Santa Maria, Cádiz, Spain; 6 Present Address: Universidad de Antofagasta, Antofagasta, Chile

## Abstract

Flatfish metamorphosis is a unique post-embryonic developmental event in which thyroid hormones (THs) drive the development of symmetric pelagic larva into asymmetric benthic juveniles. One of the eyes migrates to join the other eye on the opposite side of the head. Developmental mechanisms at the basis of the acquisition of flatfish anatomical asymmetry remain an open question. Here we demonstrate that an TH responsive asymmetric centre, determined by deiodinase 2 expression, ventrally juxtaposed to the migrating eye in sole (*Solea senegalensis*) correlates with asymmetric cranial ossification that in turn drives eye migration. Besides skin pigmentation that is asymmetric between dorsal and ventral sides, only the most anterior head region delimited by the eyes becomes asymmetric whereas the remainder of the head and organs therein stay symmetric. Sub-ocular ossification is common to all flatfish analysed to date, so we propose that this newly discovered mechanism is universal and is associated with eye migration in all flatfish.

## Introduction

Metamorphosis defines the developmental process that gives rise to a change in form. In vertebrates the best-studied examples of metamorphosis occur in anurans that change from an aquatic tadpole to a terrestrial frog. However, striking examples of metamorphosis also occur in the teleosts an infraclass of the actinopterygii that includes important sport and commercial fish^1–5^. Nowhere is metamorphosis more dramatic than in the flatfish lineage, where one eye of the pelagic pre-metamorphic symmetric larvae migrates to the opposite side of the head and generates an asymmetric benthic juvenile that tilts and settles on its blind side. Fossil evidence indicates that flatfish first arose about 50 million years ago^6^ and that eye migration was one of the earliest events in the gradual development into a fully asymmetric form.

Thyroid hormones (TH) have been demonstrated as the necessary and sufficient factors driving flatfish metamorphosis^3,7,8^. Nonetheless, the TH regulated cellular and molecular events that drive eye migration during flatfish asymmetric development remain elusive. The forces underlying flatfish eye migration have long puzzled biologists and recently it was proposed that enhanced cell proliferation in the sub-ocular region was the main cause^9^. However, the proposed model fails to explain the remodelling also encountered during metamorphosis in the ventral blind-side jaw and some eye migration that still occurs when cell proliferation is inhibited. This indicates that either, (i) cell proliferation and another unknown mechanism cause eye migration or (ii) cell proliferation is a secondary consequence of an as yet unknown mechanism that causes eye migration.

The present study on the flatfish Senegalese sole (*Solea senegalensis*) demonstrated that (besides skin pigmentation that is always asymmetric between dorsal and ventral sides) asymmetry development is restricted to a discrete region of the anterior head and that both bone and brain are remodelled. We identify for the first time at a cellular level a TH responsive asymmetric centre in the mesenchyme tissue that is correlated with ossification and associated with all the asymmetric head development observed during sole metamorphosis. This TH responsive asymmetric centre is localized on the anterior left (blind)–side of the neurocranium and is correlated with all asymmetric head development during metamorphosis in sole. In summary, we show that the driving force for eye migration is ossification specifically on the blind-side sub-optical neurocranium and is driven by THs which together with skin proliferation give rise to the observed asymmetric development of the head. As ossification progresses during metamorphosis it pushes the eye and all other optic structures already developed to the other side of the head. Our results support the hypothesis^10–15^ that the morphological changes giving rise to head asymmetry during flatfish metamorphosis are restricted to the first most rostral third of the head.

## Results

### Asymmetric development of sole head is restricted to the most anterior third

**Figure 1:**
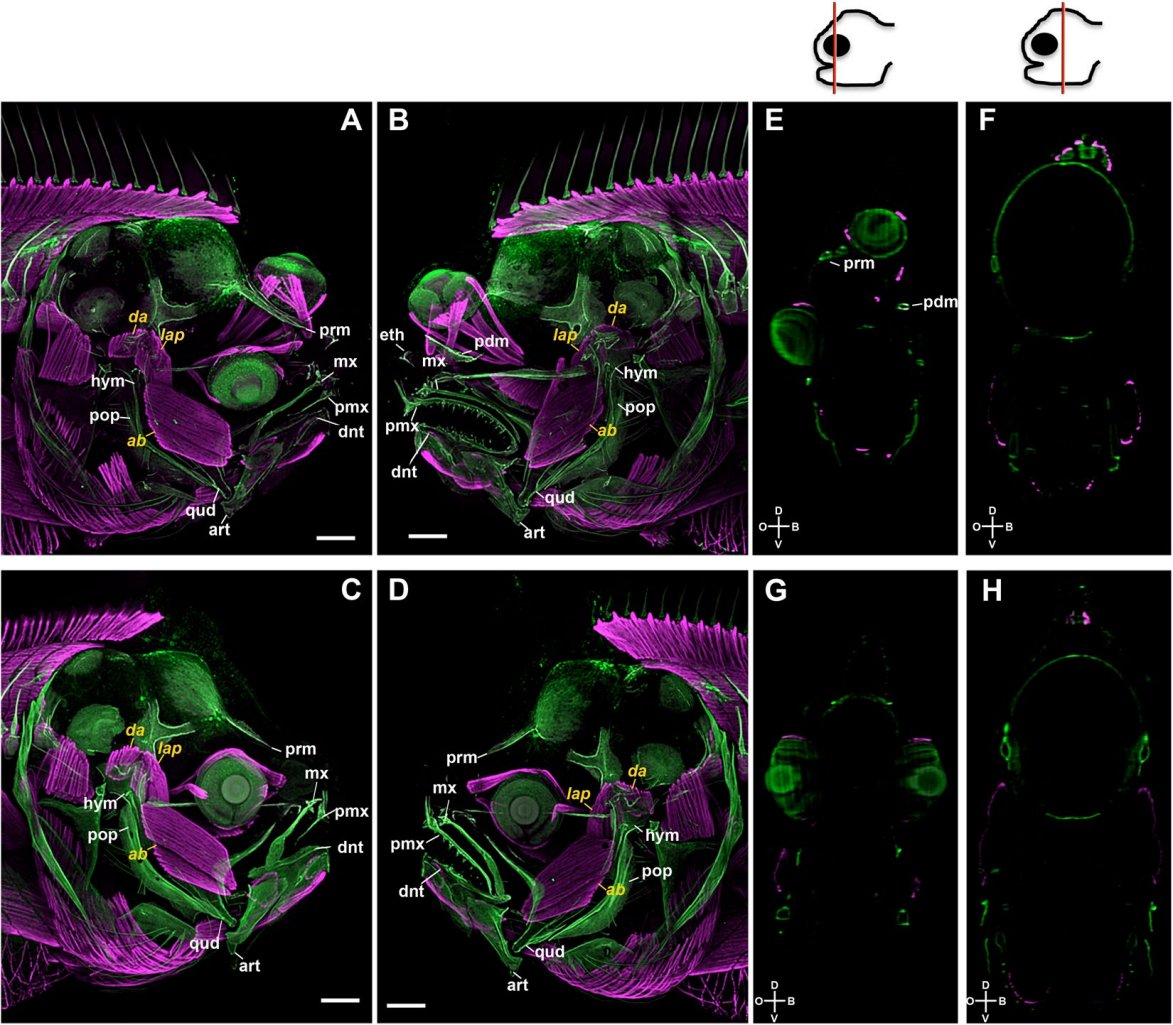
Blocking TH production with MMI inhibits asymmetric skull development of the head during sole metamorphosis. Control (**A**,**B**,**E**,**F**) and MMI-treated (**C**,**D**,**G**,**H**) larvae were labelled with osteonectin anti-serum (bone tissue, green) and anti-serum against myosin heavy chain (muscle, magenta). Maximum intensity projections of confocal microscopy stacks from ocular (**A**,**C**) or blind sides (**B**,**D**) are presented, for MMI treated and untreated (Control) fish. Transverse sections derived from orthogonal slices of reconstructed and fused whole heads of both control (**E**,**F**) and MMI-treated sole larvae (**G**,**H**). It clear in the controls that asymmetry only exists on the blind side of the larval pseudomesial (pdm) skull bone just ventral to the migrating eye. On the ocular side asymmetry is observed in the paramesial (prm) bone in (**A**,**B**,**E**,**F**). Asymmetry of the head is totally lost in MMI-treated larvae (**C**,**D**,**G**,**H**) as is asymmetry of pdm and prm development. The jaw muscles *dilator opercula* (*do*), *levator arcus palatine* (*lap*) and the *abductor mandibulae* (*ab*) are indicated in yellow. The pre-maxilla (pmx); maxilla (mx); dentary (dnt); articular (art); quadrate (qud); hyomandibular (hym); pre-opercular (pop) bones are indicated in white. Cardinal orientation denotes the dorsal (D), ventral (V), ocular (O) and blind (B) sides of the larvae. The cartoon of the head at the top of the images gives a representation of the position of the transverse head section shown in the photographic plate. Five individual larvae per treatment were imaged. Scale bars represent 200 µm.

**Figure 2:**
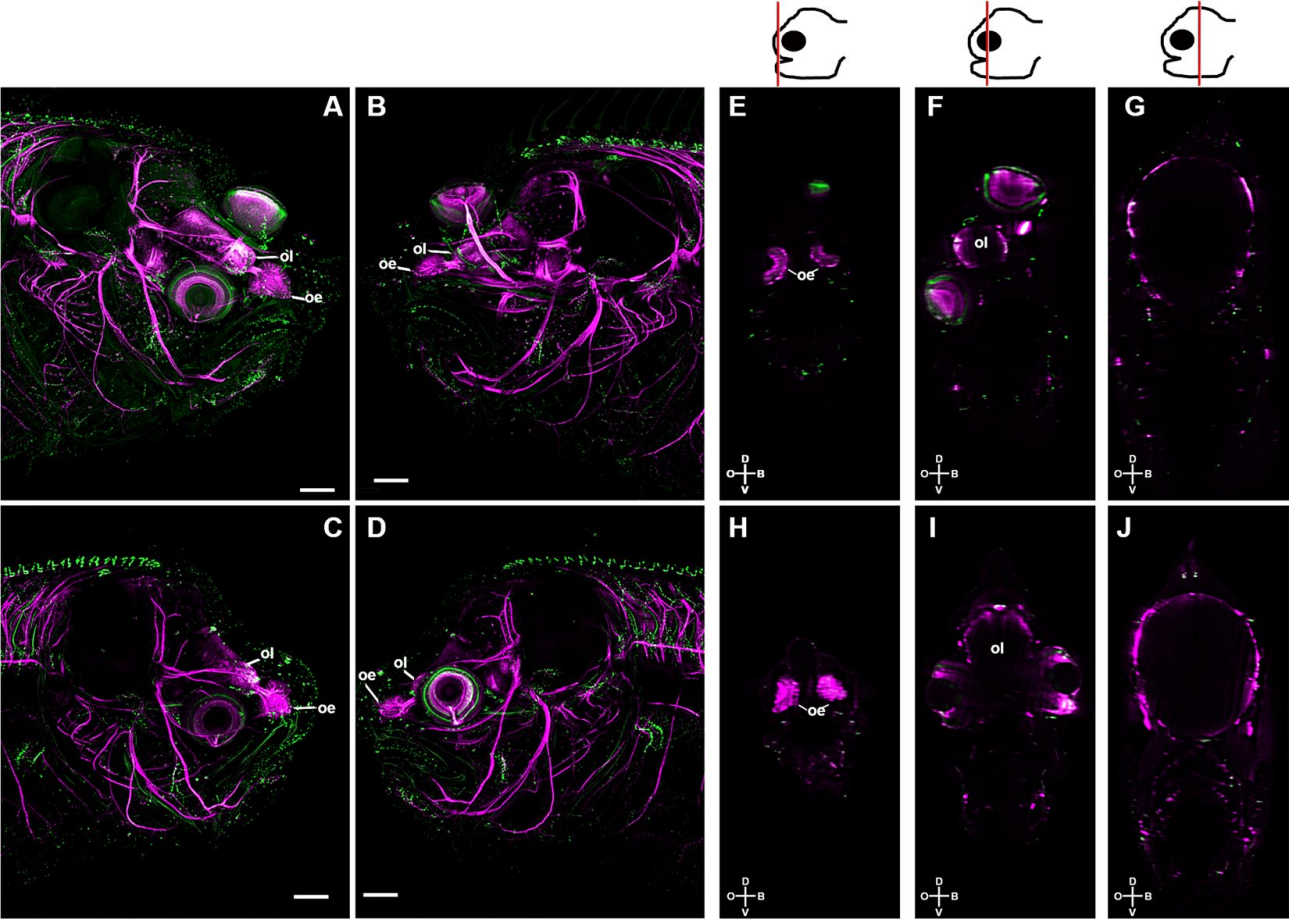
Asymmetric development of the brain during sole metamorphosis is delimited antero-posteriorly by the eyes and is blocked in the absence of TH signalling. The control at the climax of metamorphosis (S3; **A**,**B**,**E**,**F**) and the MMI-treated (**C**,**D**,**G**,**H**) larvae were labelled with anti-acetylated b-tubulin (magenta) and synaptic vesicle 2 (green). Maximum intensity projections of confocal microscopy stacks from the ocular (**A**,**B**) or blind (**B**,**D**) side of the head of control and MMI-treated sole larvae at the climax of metamorphosis are presented. Transverse sections derived from orthogonal slices of reconstructed and fused images of whole heads of control (**E**–**G**) and MMI-treated (**H**–**J**) sole larvae. Asymmetry of the neural tissues only occurs in control larvae in the region delimited by the migrating eye and includes the olfactory epithelia (oe) and bulb (ol; **A**,**B**,**E**–**G**). Conversely, in MMI-treated larvae symmetry of nervous tissue is maintained (**C**,**D**,**H**–**J**). The cartoon of the head at the top of the images gives a representation of the position of the transverse head section shown in the photographic plate. In control note the shift from asymmetry in the anterior head region to symmetry posterior to the eyes. Cardinal orientation denotes the dorsal (D), ventral (V), ocular (O) and blind (B) sides of the larvae. Five individual larvae per treatment were imaged. The scale bars represent 200 µm.

Head remodelling is the major morphological change that occurs during metamorphosis in flatfish^10–15^. For this reason, we targeted head development using whole mount immunohistochemistry for bone, muscle and nervous tissues followed by confocal microscopy and 3D reconstruction. We created a highly detailed map of the morphological changes occurring in sole head during metamorphosis (Figs 1 and 2 and Supplementary Figs 1 and 2). We found that only a small portion of the structures of the head actually became asymmetric. Interestingly, the asymmetric region was restricted to the bony and neural structures in the anterior third of the head between the snout and the end of the migrating eye field (Fig. 1 and Supplementary Fig. 1A–J,K,M,O,Q and S). Conversely, in the remainder of the post-ocular head region, the skull, and structures within, developed symmetrically (Fig. 1 and Supplementary Fig. 1,A–J,L,N,P,R and T). The migration of the eye occurred in parallel to the asymmetric development of the ethmoid (eth; anterior to the eye) and pseudomesial (pdm; medial to the eye) bones (Supplementary Fig. 1).

The asymmetry of the skull developed by mesenchyme tissue ossification in the lower quadrant of the anterior blind-side of the neurocranium (pdm in Supplementary Fig. 1). Ossification of the neurocranium proceeded in a ventral to dorsal direction and jaw remodelling occurred in a dorsal to ventral direction (Supplementary Fig. 1A,B). Simultaneously, the anterior neurocranium ossification fronts in the ocular side compressed together to build the paramesial (prm) bone between the eyes (Supplementary Fig. 1E,F and O). The blind side jaw was also remodelled (Supplementary Fig. 1A–J). The asymmetric development of the jaw was restricted to the pre-maxilla (pmx), maxilla (mx) and dentary (dnt) bones. We observed that the posterior end of these structures migrated ventrally acquiring a downward bow like morphology (Supplementary Fig. 1A–J). The development of the blind-side jaw muscles accompanied the asymmetric development of the jaw (Supplementary Fig. 1). Notably, the articular (art), quadrate (qud), hyomandibular (hym) and pre-opercular (pop) bones all developed symmetrically (Supplementary Fig. 1A–J).

The *dilator opercula* (*do*), *levator arcus palatine* (*lap*) and the *abductor mandibulae* (*ab*) jaw muscles on the blind side did not change their position or shape relative to the homologue muscles on the ocular side (Supplementary Fig. 1A–J). The ocular muscles of the blind side eye did not change their position or shape and migrated as a unit with the eye to the contralateral side of the head (white asterisks in Supplementary Fig. 1A,B).

The results show that the asymmetric development of the brain during metamorphosis was restricted to the olfactory epithelia (oe) and lobes (ol; Fig. 2 and Supplementary Fig. 2). During metamorphosis the ocular olfactory epithelia assumed a prominent cup-like shape whereas the blind-side epithelia was more open and planar (Supplementary Fig. 2A–J,K,N,Q,T and W). Interestingly, the only brain regions that became asymmetric during metamorphic development of the sole head were the olfactory lobes. They were pushed and rotated along the LR axis onto the ocular side (Supplementary Fig. 2). The proximal and distal ends of the facial nerves retained symmetry in the sole head after metamorphosis (Supplementary Fig. 2,A).

Blocking TH production (with methimazol, MMI) confirmed that the asymmetric metamorphic development of the head, muscles and nervous tissue was strictly dependent on THs (Figs 1 and 2 and Movies 1–4).

### Asymmetric TH responsiveness correlates with asymmetric development of the ethmoid and pseudomesial bones in sole head

**Figure 3:**
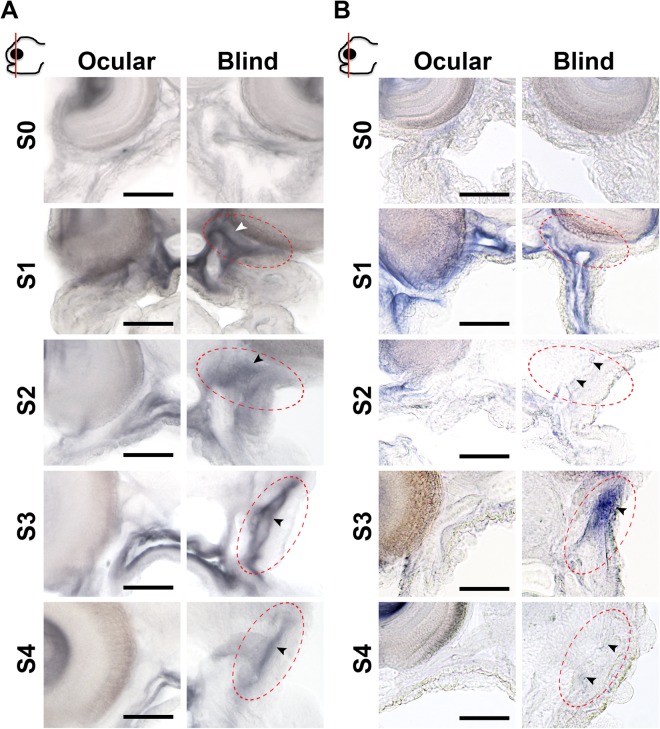
*Deiodinase 2* and *thrb* asymmetric expression determines an TH-responsive asymmetric centre localized just ventral to the migrating eye in sole. Details of the *in situ* expression of *dio2* (**A**) and *thrb* (**B**) in the sub-ocular region of the ocular and blind side (encircled red areas) of sole larvae undergoing normal metamorphosis. Arrowheads in the encircled red area highlight the asymmetric expression of *dio2* or *thrb*. Images correspond to a higher magnification of the regions in the dashed black boxes indicated in the images of the vibratome sections shown in Supplementary Figs 7(A) and 8(B). The scale bar represents 100 µm.

Cellular signalling activated by TH is tightly regulated. The key regulators are monocarboxylic transporter 8 (*mct8*), the deiodinases 2 (*dio2*) and 3 (*dio3*) and the thyroid hormone receptors, alpha a (*thraa*), alpha b (*thrab*) and beta (*thrb*)^16,17^. We mapped the expression of these genes during metamorphosis, and our results showed that most tissues in the head have the necessary signalling machinery to respond to THs (Supplementary Figs 3–8). TH signalling genes that were involved in symmetric development in sole head during metamorphosis were found in skin, brain and muscle (Supplementary Figs 3–8). Importantly, asymmetric expression of TH signalling genes was only detected for *dio2* and *thrb* genes, which had a left-right asymmetry (L-R; blind-ocular) in the sole head (Fig. 3 and Supplementary Figs 7 and 8).

**Figure 4:**
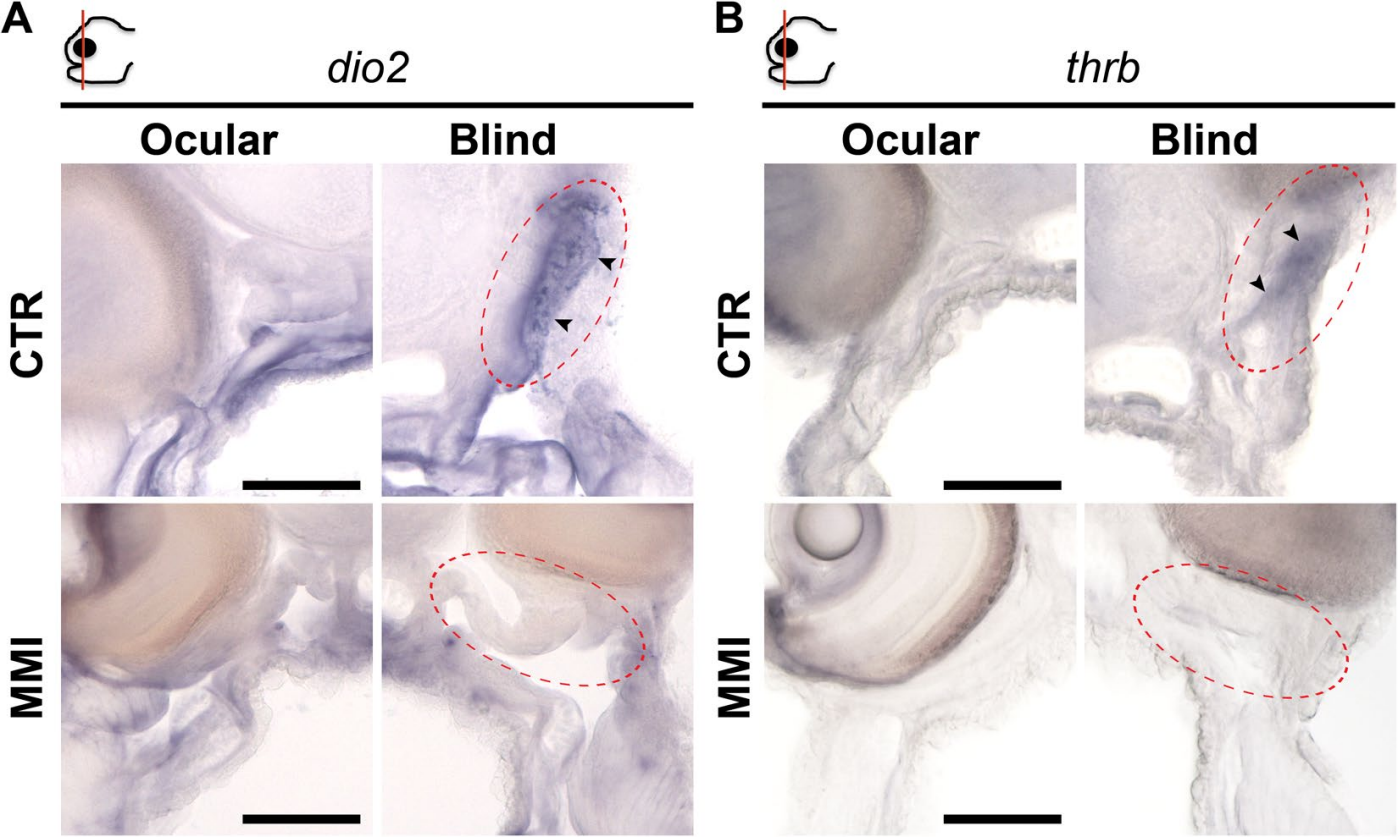
The blind side sub-ocular asymmetric centre defined by *dio2* and *thrb* is dependent on THs. MMI-treatment prevents asymmetric *dio2* (**A**) and *thrb* (**B**) expression (arrowhead in encircled red areas) in the sub-ocular blind side of sole larvae. Images correspond to a higher magnification of the regions in the dashed black boxes indicated in the images of the vibratome sections shown in Supplementary Fig. 11. The scale bar represents 100 µm.

The asymmetric expression of *dio2* and *thrb* was located in: (1) the anterior lower left quadrant of the neurocranium just ventral to the migrating eye and dorsal to the jaw, (2) stretched from the snout to the mesenchyme tissue separating the jaw muscles from the skin and (3) coincided with the head region where asymmetric development of bone and brain occurred (Figs 1 and 2 and arrowheads in encircled red areas in Fig. 3A,B and Supplementary Figs 7 and 8). Expression of *dio2* achieved an asymmetric distribution earlier than *thrb*, being clearly detectable at S1 (arrowhead in encircled red areas in Fig. 3 and Supplementary Figs 7 and 8). In turn *thrb* asymmetric expression became more evident from S2 onwards (arrowhead in encircled red areas in Fig. 3 and Supplementary Figs 7 and 8). When metamorphosis was blocked with a goitrogen (MMI) to inhibit TH production by thyrocytes, the asymmetric centre of TH-responsiveness in the head was ablated (defined by asymmetric *dio2* and *thrb* expression) (Fig. 4). This shows that TH is needed for the establishment of the hormone responsive asymmetric centre in sole head. Furthermore, the symmetric developing tissues in the head (e.g. skin, mid-brain) had enhanced *dio2* expression (Fig. 4A, Supplementary Fig. 11,A–D,I–N) while the inverse was observed for *dio3* and *thrb* (Supplementary Figs 9,A–D,I–N, 4B and Supplementary Fig. 11,E,F,O–T). The inhibition of metamorphosis by MMI demonstrates that THs positively regulate asymmetric *dio2* expression rather than negatively as observed in other head tissues where *dio2* is symmetrically expressed. As foreseen based on gene expression patterns during normal metamorphosis, *mct8*, *thraa* and *thrab* were not affected by MMI-treatment (Supplementary Figs 9,E,F,O–T and 10). These results were also confirmed by RT-qPCR performed on whole-heads: (1) expression of *dio2* was unchanged most likely given the heterogeneous response of *dio2* expression (increased in some tissues decreased in the asymmetric centre), (2) *dio3* and *thrb* were significantly down-regulated, and (3) no significant changes in the expression of *thraa*, *thrab* and *mct8* were found (Supplementary Figs 10A–F and 13).

**Figure 5:**
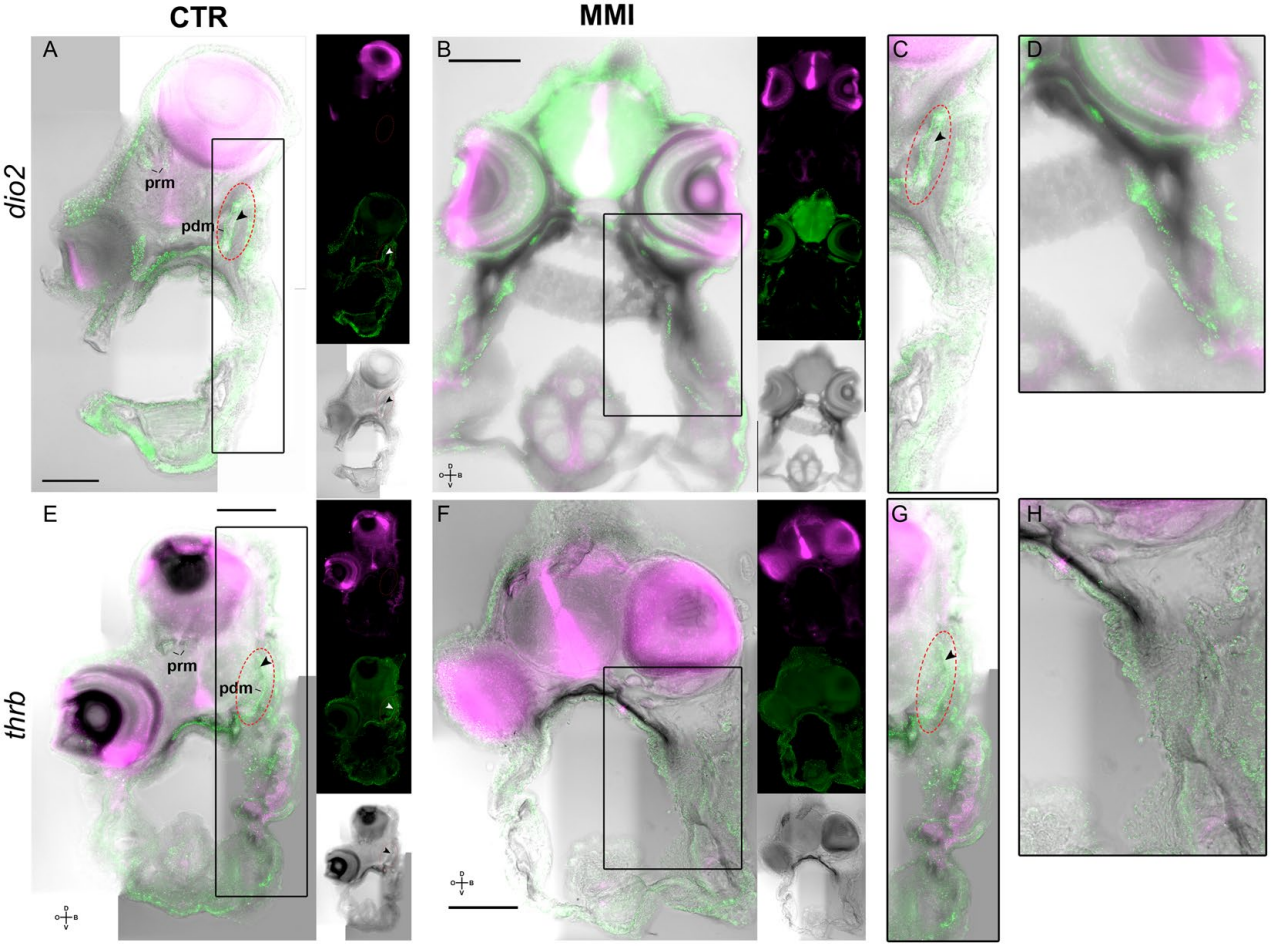
Thyroid dependent asymmetric expression of *dio2* and *thrb* demarks a TH-responsive asymmetric centre responsible for bone ossification, eye migration and asymmetric head development during sole metamorphosis. Asymmetric expression of *dio2* (**A**,**C**) and *thrb* (**E**,**G**; dark stain) is adjacent to OSN immunodetected signal (green, in extracellular matrix) in the developing pseudomesial bone (pdm) but not PCNA (magenta) in normal metamorphosing sole larvae. Note that the PCNA signal is always juxtaposed distal to the *dio2*, *thrb* and OSN signal (arrowheads in **A**,**E**,**C**,**G**). The use of MMI to block metamorphosis impairs the development of the TH-responsive asymmetric centre that is demarked by the expression of *dio2* and *thrb*. In MMI-blocked metamorphosis asymmetric *dio2* (**B**,**D**) and *thrb* (**F**,**H**) expression is lost, asymmetric bone development is not observed and OSN asymmetric expression does not occur in larvae (**B**,**D**,**F**,**H**). Blind side asymmetric PCNA expression is also lost (**B**,**D**,**F**,**H**). Although the OSN asymmetric expression is lost, symmetric expression in the skin is maintained (**B**,**D**,**F**,**H**). Likewise, symmetric PCNA expression is also maintained in the brain and eyes (**B**,**D**,**F**,**H**). Transverse vibratome sections of 3 larvae per treatment were used. All scale bars represent 200 µm. Cardinal orientation denotes dorsal (D), ventral (V), ocular (O) and blind (B) sides of the larvae.

**Figure 6:**
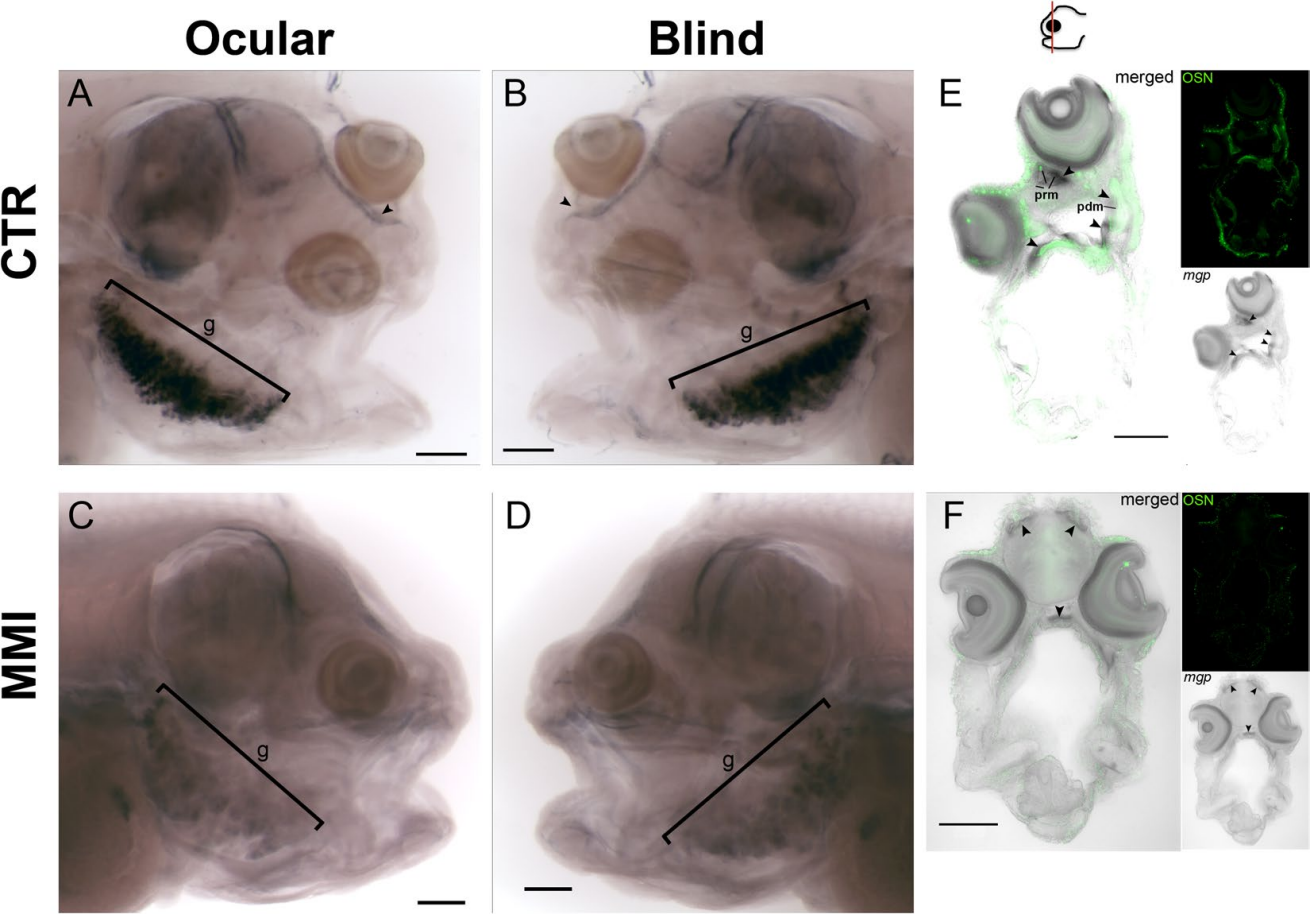
Different genetic programs regulate symmetric and asymmetric head bone development. Paramesial bone (prm) development is regulated by MGP (Matrix Gla-Protein) under the influence of THs in metamorphosis (**A**,**B**,**E**) but lost in MMI-treated larvae (**C**,**D**,**F**). However, symmetrically developing bones do not lose MGP expression in MMI-treated larvae. In the gills of normal metamorphosing sole expression of MGP is much higher than in MMI larvae but it is not lost in the latter larvae (**A**–**D**). Ten larvae per group were used in *mgp* WISH. Three individual larvae per group were used for transverse vibratome sections and OSN IHC. In images (**A**–**D**) black bracket denotes the gills(g). Scale bars represent 200 µm. Cardinal orientation denotes dorsal (D), ventral (V), ocular (O) and blind (B) sides of the larvae.

The asymmetric centre of TH signalling that included the expression signals of *dio2* and *thrb* was surrounded by an osteonectin (matrix localised) positive signal. This observation suggested a link between the TH responsive asymmetric centre and ossification of blind side bone (ethmoid and pseudomesial) (arrowheads in Fig. 5). Blocking TH production with MMI also induced loss of expression of osteonectin immunoreactivity and matrix-gla protein mRNA (MGP). We observed that the expression of these proteins was associated with the *dio2*-*thrb* TH responsive asymmetric centre in the sub-ocular and ocular region of prm; therefore our results strongly suggests that these ossifying sites are under the regulation of THs (arrowheads in Figs 5 and 6).

We propose that in sole head there are two mutually exclusive thyroid-driven ossification programs that are mediated by *dio2* and *thrb* (Fig. 5 and Supplementary Figs 7,8 and 11). Expression patterns for MGP and osteonectin differed depending on the tissue and its localization in the head during sole metamorphosis (Figs 5 and 6). Blocking of THs endogenous production prevents the expression of osteonectin located in ossifying tissue of the responsive asymmetric centre but not in the skin and MGP expression in the neurocranium but not in the gills (arrowhead in encircled red area Fig. 5 and arrowheads in Fig. 6). Our results reinforced the hypothesis that THs have a context-specific action and likely promote asymmetric ossification of the ocular side (prm), and blind side (eth and pdm) bones and that this ultimately leads to eye migration and lower jaw remodelling.

### Sub-ocular ossification leads to eye migration and head asymmetry

**Figure 7:**
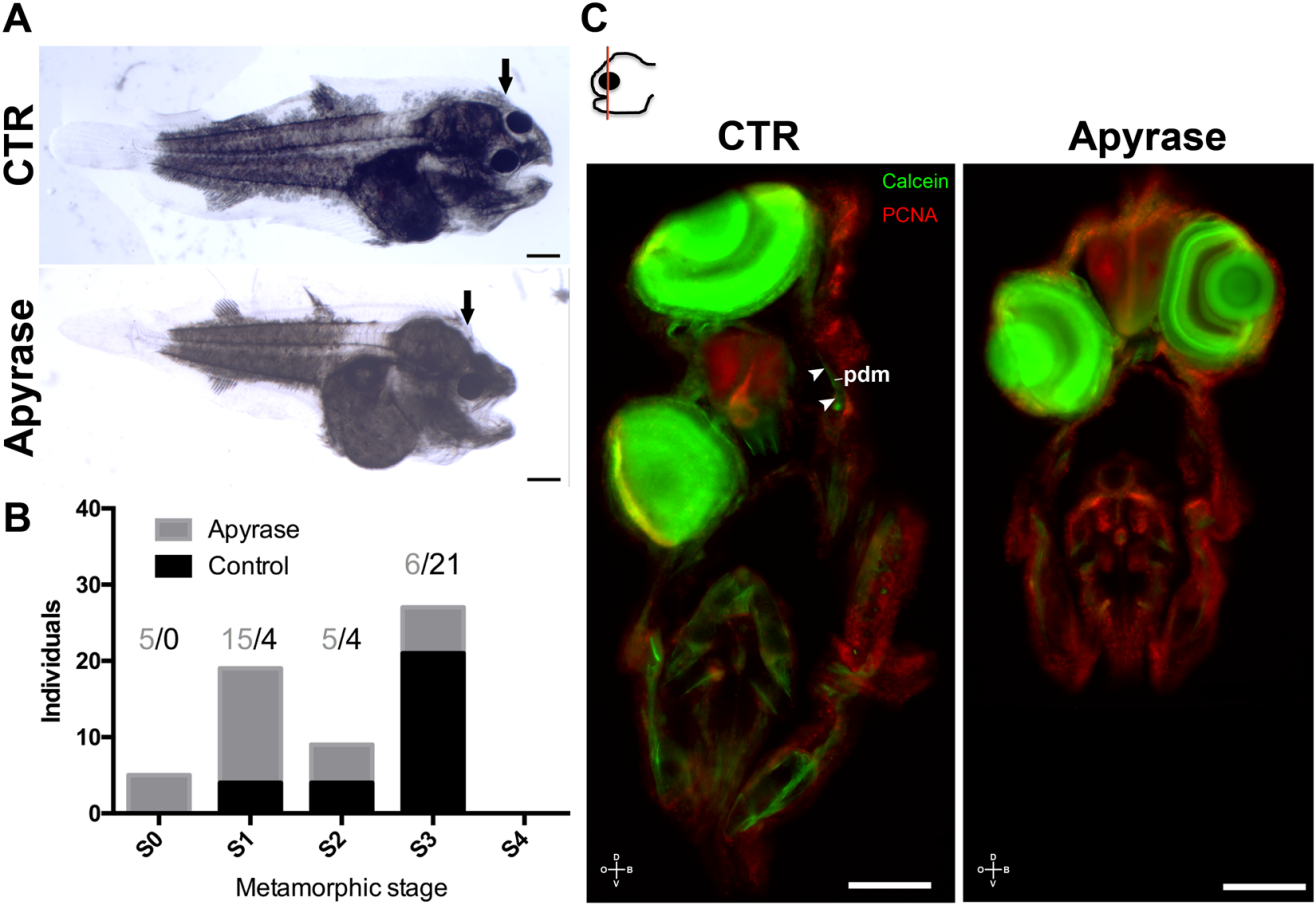
Apyrase blocking of pseudomesial ossification leads to impaired eye migration during sole metamorphosis. (**A**) Representative larvae from control and apyrase experimental groups. (**B**) Distribution of the numbers of treated larvae relative to the control larvae according to their eye migration index is indicated. Contingency analysis on trends gave highly significant differences between the treatments (Chi-square p < 0.0001). (**C**) Vibratome sections of the experimental larvae treated with apyrase and then the bone labelled with calcein (green; arrowheads denote the developing prm bone) and immunohistochemistry for PCNA (red, n = 5; **C**). In A black arrow denotes metamorphic dependent anterior expanding epaxial muscle. In C cardinal orientation denotes dorsal (D), ventral (V), ocular (O) and blind (B) sides of the larvae. The scale bar represents 100 μm.

To test the hypothesis that sub-ocular ossification leads to eye migration and head asymmetry, metamorphosing sole larvae were treated with apyrase, a compound that prevents heterotypic dermal ossification in humans by inhibiting BMP signalling^18^. In the majority of larvae in the apyrase-group, eye migration was impaired and did not reach the position expected at metamorphic climax. Nonetheless, other indexes of metamorphic progression like anterior expansion of the epaxial musculature still occurred as well as increased axial muscle height. In fact, when apyrase was injected under the “future” migrating eye only 10 out of 30 larvae had the migrating eye in the expected positon at climax, whereas in controls 21 out of 29 larvae had eyes in the expected climax position (Chi-square for trend p < 0.0001; Fig. 7). Notably, even though apyrase treated larvae exhibited metamorphosis associated changes in some of their structures they were smaller than the control larvae. The differences detected cannot be fully explained by the apyrase treatment since it was applied only to the sub-ocular region of the future blind side. One possible explanation for the growth phenotype of the apyrase-treated larvae is that their misplaced eye made it more difficult to capture prey leading to reduced size relative to the control larvae.

Interestingly, PCNA immunofluorescence revealed an increase in cell proliferation on the blind side ventral to the eye in both the control and apyrase group (Fig. 7C). Most of apyrase treated larvae had the migrating eye in the S0 or S1 stage position (none or slight eye migration), while the majority of the saline injected larvae were in the S2 or S3 stage (Fig. 7A,B). A slight eye migration was still detected in the apyrase treated larvae and is likely due to sub-epidermal cell proliferation detected by PCNA immunofluorescence. Calcein staining was used to further analyse the ossification state of the apyrase-injected larvae. We were unable to detect ossification under the migrating eye in apyrase injected-larvae whereas in saline injected-larvae a normal calcein staining signal was found indicating normal pdm bone development (arrowheads in Fig. 7C). Therefore, we conclude that apyrase inhibited sub-ocular ossification and the results confirmed our working hypothesis that eth and pdm ossification leads to eye migration and head asymmetry.

## Discussion

Collectively, our results demonstrate that the anterior region of the head (in between the snout and the posterior end of the eye field) is remodelled during metamorphosis of sole larvae. The rest of the head develops symmetrically. We identified a TH- responsive asymmetric cellular centre defined by mapping key regulators of TH signalling, *dio2* and *thrb*, which specifies ossification in the blind side of the head. The *thrb* was positively regulated by THs in both symmetric and asymmetric metamorphosing tissue whereas *dio2* expression was under the classical TH negative feedback loop in symmetrical developing tissues^19^. Conversely, in the TH-responsive asymmetric cellular centre in the sole head *dio2* expression was positively regulated by THs and this seems to be a flatfish specific adaptation. In fact, in all vertebrates the only other cells that present this type of *dio2* gene regulation by THs occurs in pituitary thyrothrophs. The expression of all other TH signalling genes was symmetric. We therefore conclude that *dio2* and *thrb* are pivotal TH-signalling genes that determine the asymmetric development of sole head during metamorphosis. Blocking metamorphosis with MMI allowed us to further conclude that asymmetric development of head bones was correlated with asymmetric TH-responsivity. Taken together, the reported results indicate that during metamorphosis different types of TH-dependent developmental programs exist that share the classical players of TH-cellular signalling but that responds to THs in a different way. The factors underlying this complex regulation are still unknown.

It has previously been proposed that the remodelling of the anterior skull may be the driving force behind asymmetry in odorant sensing in post-metamorphic juvenile sole^20^. We hypothesize that the development of asymmetric morphology may explain the asymmetric olfactory function. Very importantly, none of the other regions of the brain analysed displayed morphological asymmetry. Nonetheless, given that we mapped genes restricted primarily to TH-signalling we cannot exclude the existence of functional asymmetry in other regions of the brain.

It has been proposed that cell proliferation of the dermal tissue ventral to the migrating eye is responsible for asymmetric development of the head in flatfish^9^. However, our data showed that although cell proliferation contributes to eye migration it was not the primary driving force of asymmetric development in the flatfish head during metamorphosis. This notion was supported by the observation that inhibition of blind side ossification with apyrase blocked eye migration, even though cell proliferation still occurred in both dorsal and ventral fronts of the asymmetric centre in both treated and untreated larvae.

**Figure 8:**
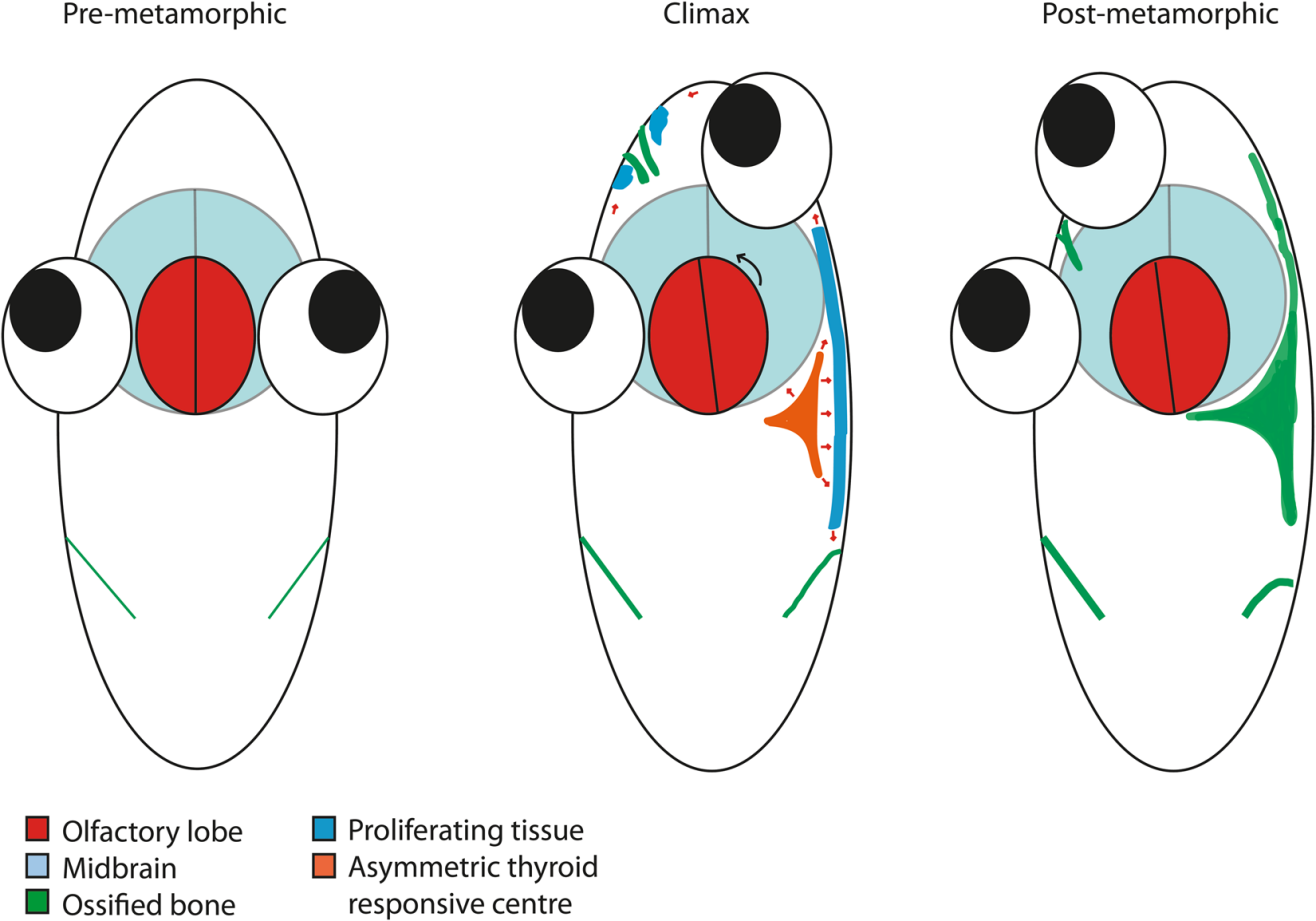
Proposed model summarizing the mechanisms that drive flatfish eye migration during metamorphosis. In pre-metamorphic larvae (left panel) all the head tissues are symmetrical. As metamorphosis progresses and climax is reached (middle panel) the TH-responsive asymmetric centre, which is demarked by *dio2*, *thrb* and osteonectin expression arises and causes asymmetric bone development that together with increased cell proliferation in distal adjacent tissue leads to eye migration and jaw remodelling. In the proposed model these combined events lead to passive blind-side eye migration which compresses the frontal portion of the neurocranium. The neurocranium then fuses to give rise to the paramesial bone in-between the eyes. Cell proliferation that delimits the fusing neurocranium elements gives rise to the paramesial bone. After metamorphosis (right panel) the TH-responsive asymmetric centre has developed into the pseudomesial and ethmoid bones on the blind side (now ventral) and the paramesial bone has fully fused. No cell proliferation is observed after metamorphosis.

The results of our study show that: (1) ossification of eth and pdm in the blind side of metamorphosing sole larvae is the main driver of eye migration; (2) THs are correlated with both ossification and increased dermal proliferation in the blind sub-optical side of metamorphosing sole larvae (Fig. 8); (3) asymmetric expression of TH-signalling mediator genes *dio2* and *thrb* was strongly correlated in time and in space with asymmetric ossification strongly suggesting that THs are the driving force behind this developmental event; (4) ossification and dermal cell proliferation in the blind side are two independent developmental events promoted by THs; and (5) the THs are integrators that coordinate bone ossification and cell proliferation during eye migration. Our data further indicates that asymmetric ossification in the head increases the density of the sub-dermal tissue and we hypothesize that as a consequence dermal tissue proliferates to accompany the growth of the enlarging underlying ossified tissue leading to eye migration (Fig. 8).

In conclusion, we provide for the first time evidence of a TH-responsive asymmetric centre located in the anterior head region that is correlated with asymmetric development during metamorphosis of flatfish. Moreover, migration of the eye, remodelling of the jaw and olfactory epithelia and lobes are a consequence of asymmetric bone development. Sub-ocular ossification is common in all flatfish studied to date, hence our results unmask a previously uncharacterised mechanism for post-embryonic asymmetric development. We propose that this is a universal mechanism that drives eye migration in all flatfish.

## Materials and Methods

### Sole rearing and sampling during normal metamorphosis

All sole cultures were carried out as described previously^21^. Pre-metamorphic (S0), onset (S1), early climax (S2), climax (S3) of metamorphosis and post-metamorphic juvenile (S4) samples were collected using the established staging scheme^22^. A sub-sample of larvae were collected and transferred to 500 mL of sterile seawater with 0.2% calcein (Sigma; pH7) and left for 15 minutes at room temperature. After calcein treatment larvae were rinsed 5 times in sterile seawater, anesthetized with 0.125 mg/L of MS222 (Sigma) and fixed for 1 hour at room temperature in 1% PFA/1xPBS(pH7.4). The remaining larvae from cultures were fixed overnight at 4 oC in 4% PFA/1 x PBS (pH 7.4). After fixation all larvae were washed 4 × 10 minutes in 1 x PBS (pH7.4) + 0.1% tween 20 (PBT) and de-pigmented in 1 x PBS/0.5% KOH/3.5% H_2_O_2_ at room temperature. After de-pigmentation larvae were transferred to a graded series of 1x PBS/methanol (50 → 100%) and stored in methanol at −20 C until use.

All experiments were carried out in accordance to Portuguese, Spanish and EU law for animal well fare and approved by the ORBEA-Animal Welfare Committee of CCMAR and IFAPA.

### Methimazol blocking of metamorphosis

Blocking of metamorphosis with methimazol (MMI) was carried out using 0.3 mM MMI dissolved in seawater as described previously^21^. Samples were collected 13 days after initiation of the treatment when control larvae where at S3. Larvae were fixed in 4% PFA/1 x PBS overnight and processed as described above.

All experiments were carried out in accordance to Portuguese, Spanish and EU law for animal well fare and approved by the ORBEA-Animal Welfare Committee of CCMAR and IFAPA.

### Sea bream osteonectin (saOSN) antisera production and validation

The mature coding region of sea bream (*Sparus aurata*, sa) OSN (accession number AJ564190) was cloned in frame with the N-terminal tag of the T7/NT-TOPO expression vector (pCR T7 TOPO TA Expression Kits, Invitrogen, USA) following the supplier’s instructions. The fusion protein, His-tagged sea bream OSN (His-saOSN), was expressed in *E. coli* BL21 (DE3)plysS (Invitrogen), purified by continuous elution electrophoresis using a Model 491 Prep Cell (Bio-Rad, Portugal)^23^ and its authenticity confirmed by mass spectrometry analysis (MALDI-TOF). A polyclonal antisera was produced in rabbits against purified His-saOSN (STABvida, Portugal) and the saOSN antisera specificity was validated by Western blot. Purified His-saOSN (0.5 μg per well) was fractionated by SDS-PAGE in three subsequent wells (12% polyacrylamide gel) and blotted onto a hydrophobic polyvinylidene difluoride membrane (PVDF, Hybond-P, Amersham GE Healthcare, UK) for 1 h at 300 mA in a vertical tank transfer system (TE 22 Mighty Small Tank transfer, Hoeffer, Amersham GE Healthcare, US). After blotting the PVDF membrane was cut and each of the well-run was incubated with either saOSN antisera (1/50,000; 1 in Supplementary Fig. 14B), pre-immune sera (1/50,000; 2 in Supplementary Fig. 14B) or saOSN antisera (1/50,000) pre-absorbed with recombinant His-saOSN (3 in Supplementary Fig. 14B) and detected using peroxidase labelled secondary antibody using the method described in^23^. Membranes were developed with an ECL Plus Western Blotting Chemiluminescent Detection kit (Amersham GE Healthcare, UK, Supplementary Fig. 14B) and exposed for 5 minutes to photographic paper (Kodak) in the dark. In parallel it was ran a similar gel that was used for Commasie blue (Supplementary Fig. 14A) to ensure equal loading in Western blot analysed membrane. The working dilution of the His-saOSN antisera was optimized in a preliminary study^23^.

### Whole-mount immunohistochemistry

Larvae (n = 5/stage/staining) were hydrated through a graded series of methanol/PBS and rinsed several times in 1xPBS/0.1% Triton-X100 (PBTr, Sigma). Tissue permeabilization was carried out with 5 μg/mL collagenase (Sigma) in 1xPBS at room temperature. The larvae were re-fixed in 4% PFA/1xPBS (pH7.4) and washed several times in PBTr at room temperature. Incubation was carried out in 1xPBS/10% sheep serum/0.1% triton containing an appropriate concentration of primary antisera. Striated muscle labelling was carried out with mouse MF20 or A4.1025 (DSHB) at 5 μg/mL; mouse anti-acetylated β-tubulin antiserum (2 μg/mL; AA2; Sigma) and mouse anti-synaptic vesicle glycoprotein 2A (2 μg/mL; SV2; DSHB) were used to label neural tissue. The saOSN antiserum (1/5000) was used to detect developing bone. Detection was carried out using a goat secondary antiserum against mouse IgG H + L conjugated with Hilyte 594 dye (1/400; Anaspec) and/or goat anti-mouse IgG2gama conjugated with Alexa-488 dye (1/400; Invitrogen). For detection of OSN a goat-anti rabbit secondary antiserum conjugated with Hilyte-488 was used (1/400; Anaspec). Samples were clarified with the ScaleA2 solution using the established methodology^24^ and transferred to glycerol for imaging in a Zeiss LSM710 confocal microscope or in a Zeiss Z2 fluorescent light microscope.

### Whole-mount *in situ* hybridization

Expression analysis by WISH was carried out as previously described^21^ (n = 10 individual larvae/gene/stage or treatment). Riboprobes for *dio2*, *dio3*, *mct8*, *thraa*, *thrab* and MGP were prepared after PCR cloning into the pGemT-easy vector (Promega) and PCR amplification (Primers in Supplementary Table 1). The riboprobe for *thrb* was prepared from a previously isolated clone^7^. PCR products were used to prepare sense (Supplementary Fig. 12) and anti-sense RNA probes using T7, T3 or SP6 RNA polymerases as appropriate and DIG labelling mix (Roche).

### Vibratome sectioning and immunohistochemistry

Vibratome sectioning of WISH analysed larvae (n = 3/stage or treatment) was carried out as previously described^21^. Floating section immunohistochemistry against osteonectin and PCNA was carried out simultaneous using a 1/5000 dilution of rabbit saOSN polyclonal antisera and a 1/200 dilution of mouse monoclonal serum against PCNA (Dako). Detection was carried out simultaneously for both antiserums using respectively, goat-anti rabbit secondary antiserum conjugated with Hilyte-488 (1/400; Anaspec) and goat anti-mouse secondary antiserum conjugated with Hilyte-594 (1/400; Anaspec). Sections were placed on glass slides and Z-stacked images taken using a Zeiss Z2 fluorescent microscope.

### Image acquisition and processing

For larvae processed by whole mount immunohistochemistry, each side of the head was imaged separately using confocal microscopy on a Zeiss LSM710 system using a 10 × 0.2NA LENS. For each larvae two 3D stacks were acquired, one from the ocular, and another from the blind side. The datasets were then deconvolved using Huygens 2.3 software (SVI) and the two halves fused into a single 3D dataset using Amira 5.3 software (FEIa). 3D animations were prepared using the software Amira v5.3. Before rendering the animations, we processed the stitched ocular and blind side datasets to enhance the contrast of anatomical structures; for “muscle” we subtracted the green fluorescence (bone/OSN staining) from the A4. immuno-staining signal (there was noticeable crosstalk between the two channels), and for “nerves” we enhanced the signal of bTub by applying a gamma followed by a top-hat filter. A top-hat filter was also applied to the synaptic vesicle staining. Gray-scale images of “background tissues” were obtained by applying to the green fluorescence dataset a gamma filter and a large open morphological operator. Further image analysis was carried out in FIJI^25^. For vibratome sections images captured in different fluorescent channels were deconvoluted in Huygens 2.3 (SVI). When necessary slice images were stitched together using the stitching plug-in^26^ in FIJI^25^.

### qPCR expression analysis

Primers for sole *dio2*, *dio3*, *thraa*, *thrab*, *thrb* and *mct8* were designed using Primer3 software (Supplementary Table 2). Preliminary assays were carried out to define optimum annealing temperatures and single product amplification. Total RNA extraction, DNase and first strand synthesis from microdissected head pools in control and MMI-treated animals was carried out as previously described (n = 5/treatment group)^1^. Amplified bands were isolated from agarose gel using a GFX gel extraction kit (GE Healthcare) and sequenced using Big Dye chemistry. Amplicon identity was confirmed by tBLASTx analysis^27^. Isolated bands were quantified in a Nanodrop spectophometer and used to prepare standard curves. qPCR expression analysis was carried out using EVAGREEN qPCR master mix (1x; Bio-Rad) with 300 mM of each primer in an Applied Biosystems One-Step plus thermocycler with the following program: 5 minutes at 95 °C followed by 45 cycles of 10 seconds 95 °C, 20 seconds at 60 °C, 10 seconds at 72 °C followed by a melt-curve analysis from 70–95 °C. Teleostean qPCR 18 s primers^12^ were used to amplify 18 s that was used as the reference gene.

### Apyrase blocking of ossification

Sole embryos were prepared as previously described until pre-metamorphic larvae. At this time larvae were transported from El Toruño research station (El Puerto de Santa Maria, Spain) to CCMAR facilities (Faro, Portugal). Larvae were acclimated for 2 days in 70 L conical tanks (50 larvae/L; 17 C) and fed normally for this age. Approximately 100 larvae were collected for treatments, anesthetized with MS222 (0.125 mg/L; Sigma) and microinjected, using an air-pressured microinjecter, with either 1XPBS + 0.1% phenol red (Control group; n = 44) or 1xPBS with 1 mg/uL apyrase (~10 U/uL; Sigma) + 0.1% phenol red (n = 50). Microinjection was given immediately ventral to the migrating eye and dorsal to the jaw every 3 days over a period of 12 days (4 injections in total for surviving larvae). After injection larvae were put in a 1 L recipient with well-aerated seawater before being put in individual 70 L conical tanks. Experiment was stopped when control larvae reached metamorphic climax (S3) and larvae (n, control = 29; n, Apyrase treated = 31) transferred to 500 mL of sterile seawater with 0.2% calcein (Sigma; pH7) and incubated 15 minutes at room temperature. Experimental larvae were washed 5 times in sterile seawater, anesthetized in 0.125 mg/mL MS222, scored for metamorphic index and photographed on a SZX7 using an Optica 3 digital camera before they were fixed overnight at 4 °C in 4% PFA/1xPBS for subsequent PCNA immunohistochemistry as previously described. The effect of apyrase treatment on metamorphic progression was measured as eye migration. Statistical analysis of the results was carried out using a contingency analysis on trends (Chi-square for trends) using Prism GraphPad6.

All experiments were carried out in accordance to Portuguese, Spanish and EU law for animal well fare and approved by the ORBEA-Animal Welfare Committee of CCMAR and IFAPA.

## Electronic supplementary material

Movie 1

Movie 2

Movie 3

Movie 4

Supplementary Figures and Legends
