# Supplementary material for: A thyroid hormone regulated asymmetric responsive centre is correlated with eye migration during flatfish metamorphosis
Source: Sci Rep. 2018 Aug 16;8:12267. doi: 10.1038/s41598-018-29957-8 (PMC6095868; doi:10.1038/s41598-018-29957-8)
Supplement: Supplementary file 5 — Supplementary Figures and Legends [file 41598_2018_29957_MOESM5_ESM.pdf]

# A thyroid hormone regulated asymmetric centre drives eye migration during flatfish metamorphosis

Marco A. Campinho[1*], Nádia Silva[1], Gabriel G. Martins[2], Liliana Anjos[1], Claudia Florindo[3], Javier Roman-Padilla[1,†], Ana Garcia-Cegarra[1,¥], Bruno Louro[1], Manuel Manchado[4] and Deborah M. Power[1]

Classification: Biological Sciences, Developmental Biology

Author affiliations:

[1]- Comparative Endocrinology and Integrative Biology Group, CCMAR, Universidade do Algarve, Campus de Gambelas, 8005-139 Faro, Portugal

[2]- Instituto Gulbenkian de Ciência, Rua da Quinta Grande, 6, 2780-156 Oeiras, Portugal

[3]-CBMR, Centre for Biomedical Research, Departamento de Ciências Biomedicas e Medicina, Universidade do Algarve, Campus de Gambelas, 8005-139 Faro, Portugal

[4]- IFAPA Centro El Toruno, 11500 El Puerto de Santa Maria, Spain

[†]- Current address: IFAPA Centro El Toruno, 11500 El Puerto de Santa Maria, Spain

[¥]- Current address: Universidad de Antofagasta, Chile

Corresponding author: Marco António Campinho. CCMAR, Universidade do Algarve, Campus de Gambelas, Ed8, Lab 3.32 8005-139 Faro, Portugal. Tel. +351289800900x7375. macampinho@ualg.pt

Supplementary Figure 1. Asymmetric skull development leads to eye migration in naturally metamorphosing sole. Maximum projections of whole-mount confocal microscopy stacks from the ocular (A, C, E, G and I) and blind (B, D, F, H and J) sides of the head in normally developing sole larvae before metamorphosis (S0; A and B), at the onset of metamorphosis (S1; C and D), early climax (S2; E and F), climax (S3; G and H) and post-metamorphic juveniles (S4; I and J) labelled with calcein (to label bone tissue, green) and MF20 anti-serum (to label striated muscle, magenta). Transverse sections derived from orthogonal slices of reconstructed and fused heads of normally developing sole larvae before metamorphosis (S0; E and L), at the onset of metamorphosis (S1; M and N), early climax (S2; O and P), climax (S3; Q and R) and post-metamorphic juveniles (S4; S and T). Asymmetry is only observed on the blind side of control larvae with the development of the pseudomesial (pdm; arrow) skull bone just ventral to the migrating eye and paramesial (prm; arrow) bone on the ocular side (A, B, E and F). The cartoon of the head at the top of the images gives a representation of the position of the transverse head section shown in the photographic plate. Note the shift from asymmetry in the anterior head region to symmetry posterior to the eyes. Jaw muscles *dilator opercula* (*do*), *levator arcus palatine* (*lap*) and the *abductor mandibulae* (*ab*) are indicated in yellow. Ocular muscles are denoted by white asterisks in A and B. Pre-maxilla (pmx); maxilla (mx); dentary (dnt); articular (art); quadrate (qud); hyomandibular (hym); pre-opercular (pop) bones are indicated in white. Five animals per stage were imaged. Cardinal orientation denotes the dorsal (D), ventral (V), ocular (O) and blind (B) sides of the larvae. All the scale bars represent 200μm.

Supplementary Figure 2. Asymmetric brain development during sole metamorphosis is restricted to the olfactory lobe. Maximum projections of whole-mount confocal microscopy stacks from ocular (A, C, E, G and I) and blind (B, D, F, H and J) sides of the head in natural developing sole larvae before metamorphosis (S0; A and B), at the onset of metamorphosis (S1; C and D), early climax (S2; E and F) and climax (S3; G and H) labelled with anti-acetylated beta tubulin (magenta) and SV2 anti-serum (green). Post-metamorphic

juveniles (S4; I and J) were only labelled with acetylated beta tubulin anti-serum. Transverse sections derived from orthogonal slices of reconstructed and fused images of heads of normally developing sole larvae before metamorphosis (S0; K-M), at the onset of metamorphosis (S1; N-P), early climax (S2; Q-S), climax (S3; T-V) and in post-metamorphic juveniles (S4; W-Y). Asymmetry of the brain was only observed in the olfactory lobe (ol) and in the olfactory epithelia. The cartoon of the head at the top of the images gives a representation of the position of the transverse head section shown in the photographic plate. Note the shift from asymmetry in the anterior head region to symmetry posterior to the eyes. Five animals per stage were imaged. The cardinal orientation denotes dorsal (D), ventral (V), ocular (O) and blind (B) sides of the larvae. All scale bars represent 200μm.

Supplementary Figure 3. *dio3* is expressed symmetrically in the head of sole during metamorphosis. WISH analysis of *dio3* in sole head during metamorphosis (n=10/stage) and in the respective transverse vibratome sections (n=3/stage). WISH lateral images of sole larvae heads at premetamorphosis (S0, A and B), beginning (S1, C and D), early climax (S2, E and F) and the climax of metamorphosis (S3, G and H) and in post-metamorphic juveniles (S4, I and J). Vibratome sections of *dio3* WISH analysed larvae from pre-metamorphic (S0, K-M), beginning (S1, N-O), early climax (S2, Q-S) and the climax of metamorphosis (S3, T-V) and in post-metamorphic juveniles (S4, W-Y). The vibratome sections correspond to the snout (K, N, Q, T and W), the middle-eye field (L, O, R, U and X) and the post-eye field (M, P, S, V and Y). *dio3* is mostly expressed before metamorphosis and decreases as the larvae undergo metamorphosis. The expression of *dio3* in the head is highest in the tectum (arrowheads) and retina at S0 (A, B, L and M) but starts to decrease in early metamorphosis (S1, C, D, N-P) and early climax (S2, E, F, Q-S) but at climax (S3, G, H, T-V) it is confined to the most anterior dorsal regions of the hindbrain and dorsal posterior limit of the second brain ventricle (V). However, in S3 the retina and pituitary still has a high expression of *dio3*. After metamorphosis (S4, I, J, W-Y) expression of dio3 in the retina is mostly lost but it become evident in low abundance in the skin, the diencephalum and some regions of the anterior

hindbrain. The cartoon of the head at the top of the images gives a representation of the position of the transverse head section shown in the photographic plate. In all vibratome sections cardinal points depict dorsal (D), ventral (V), ocular (O) and blind (B) sides. In all the images the scale bar represents 100μm.

Supplementary Figure 4. The T3 specific transporter *mct8* is expressed symmetrically in the head of sole undergoing metamorphosis. WISH expression analysis of *mct8* in sole head during metamorphosis (n=10/stage) and the respective transverse vibratome sections (n=3/stage). WISH lateral images of sole larvae head in premetamorphosis (S0, A and B), beginning (S1, C and D), early climax (S2, E and F) and at the climax of metamorphosis (S3, G and H) and in post-metamorphic juveniles (S4, I and J). Vibratome sections of *mct8* WISH analysed larvae from pre-metamorphic (S0, K-M), beginning (S1, N-O), early climax (S2, Q-S) and the climax of metamorphosis (S3, T-V) and in post-metamorphic juveniles (S4, W-Y). The vibratome sections correspond to the snout (K, N, Q, T and W), middle-eye field (L, O, R, U and X) and post-eye field (M, P, S, V and Y). *mct8* is expressed symmetrically in sole larvae before metamorphosis (S0, A, B, K-M) in the otic capsule (arrowheads). Low expression is also observed in the dorsal region of the first and second brain ventricle although at lower levels. As larvae enter metamorphosis (S1, C, D, N-P) expression of *mct8* increases, particularly in the dorsal region of the mouth just below the eyes. Some expression is also observed in the frontal bones of the skull. Immediately after the eyes (P) *mct8* expression continues to be highly expressed in the otic capsule (arrowheads) and lower expression is also observed along the dorsal half of the second brain ventricle. As the larvae enter climax (S2, E, F, Q-S) expression of *mct8* is somewhat decreased. High expression is found in the otic capsule and some expression is observed in the frontal bones (Q) and second ventricle of the brain (S). At climax (S3, G, H, T-V) *mct8* expression continues in the otic capsule and is similar to previous stages and is present in the dorsal portion of the brain ventricles (U, V). After metamorphosis (S4, I, J, W-Y) asymmetric expression of *mct8* is observed at the posterior end of the eye field (arrowhead in X). After the eye field expression of

*mct8* continues in the otic capsule but is low abundance and is always symmetrical (Y). The cartoon of the head at the top of the images gives a representation of the position of the transverse head section shown in the photographic plate. In all vibratome sections cardinal points depict dorsal (D), ventral (V), ocular (O) and blind (B) sides. In all images the scale bars represent 100µm.

Supplementary Figure 5. Expression of *thraa* occurs in sole head at all stages of metamorphic development but always in a symmetric fashion and its expression is restricted mostly to the brain. WISH expression analysis of *thraa* in sole head during metamorphosis (n=10/stage) and in the respective transverse vibratome sections (n=3/stage). WISH lateral images of sole larvae heads in premetamorphosis (S0, A and B), beginning (S1, C and D), early climax (S2, E and F) and at the climax of metamorphosis (S3, G and H) and in post-metamorphic juveniles (S4, I and J). Vibratome section of *thraa* WISH in larvae from pre-metamorphosis (S0, K-M), beginning (S1, N-O), early climax (S2, Q-S) and the climax of metamorphosis (S3, T-V) and in post-metamorphic juveniles (S4, W-Y). The vibratome sections correspond to the snout (K, N, Q, T and W), middle-eye field (L, O, R, U and X) and post-eye field (M, P, S, V and Y). Expression of *thraa* is found in the most anterior diencephalon but is also present in the posterior hindbrain. Expression of *thraa* is almost not changed as the larvae undergo metamorphosis. The cartoon of the head at the top of the images gives a representation of the position of the transverse head sections shown in the photographic plate. In all vibratome sections cardinal points depict dorsal (D), ventral (V), ocular (O) and blind (B) sides. In all images the scale bars represent 100µm.

Supplementary Figure 6. Expression of *thrab* during metamorphosis of sole is mostly restricted to the brain and is always symmetrical. WISH expression analysis of *thrab* in sole head during metamorphosis (n=10/stage) and in the respective transverse vibratome sections (n=3/stage). WISH lateral images of sole larvae heads in premetamorphosis (S0, A and B), beginning (S1, C and D), early climax (S2, E and F) and the climax of metamorphosis (S3, G and H) and in

post-metamorphic juveniles (S4, I and J). Vibratome sections of *thrab* WISH analysed larvae from pre-metamorphic (S0, K-M), beginning (S1, N-O), early climax (S2, Q-S) and the climax of metamorphosis (S3, T-V) and in post-metamorphic juveniles (S4, W-Y). The vibratome sections correspond to the snout (K, N, Q, T and W), middle-eye field (L, O, R, U and X) and the post-eye field (M, P, S, V and Y). The expression of *thrab* is found in the brain in low abundance. The cartoon of the head at the top of the images gives a representation of the position of the transverse head sections shown in the photographic plate. In all the vibratome sections cardinal points depict dorsal (D), ventral (V), ocular (O) and blind (B) sides. In all images the scale bars represent 100μm.

Supplementary figure 7. WISH of deiodinase 2 (*dio2*) in sole head during metamorphosis (n=10/stage) and the respective transverse vibratome sections (n=3/stage). *dio2* is expressed symmetrically in sole head skin, muscle and brain at all stages of metamorphosis. As larvae start to undergo metamorphosis symmetric expression increases mostly in the otic capsules (arrowheads) but also in the hindbrain (M, P, S and V). As soon as larvae enter metamorphosis (S1, C, D and N-P) a new asymmetric centre of expression appears ventral to the migrating eye (red dotted circle in N, O, R, T, U, W and X). The asymmetric *dio2* expression centre is located in the mesenchyme tissue that underlies the ventral blind side quadrant of the skull and extends the entire length of the eye to the distal mesenchyme tissue underlying the lower jaw (red dotted circle in N, O, R, T, U, W and X). Asymmetric expression of *dio2* increases up until climax (S3, G, H and T-V). After metamorphosis (S4, I, J and W-Y) both asymmetric and symmetric *dio2* expression is almost lost and it is only found around the midline of the hindbrain (Y). Whole-mount lateral images of the sole larval head of pre (S0, A and B), beginning (S1, C and D), early climax (S2, E and F) and climax of metamorphosis (S3, G and H) and in post-metamorphic juveniles (S4, I and J). Vibratome sections of larvae analysed by *dio2* WISH from pre- (S0, K-M), beginning (S1, N-O), early climax (S2,Q-S) and climax of metamorphosis (S3, T-V) and in post-metamorphic juveniles (S4, W-Y). Vibratome sections correspond to snout (K, N, Q, T and W), middle-eye field (L, O, R, U and X) and post-eye field

(M, P, S, V and Y). The cartoon of the head at the top of the images gives a representation of the position of the transverse head section shown in the photographic plate. In all the vibratome sections cardinal points depict dorsal (D), ventral (V), ocular (O) and blind (B) sides. In all images scale bars represent 100µm.

Supplementary Figure 8. WISH expression analysis of thyroid receptor beta (*thrb*) in sole head during metamorphosis (n=10/stage) and the respective transverse vibratome section (n=3/stage). *thrb* is expressed symmetrically in sole head muscle and brain at all stages of metamorphosis, most notably in the otic capsules (arrowheads) and ventral region of the hindbrain. As larvae start to undergo metamorphosis symmetric expression increases. As soon as the larvae enter metamorphosis (S1, D, E and N-P) a new asymmetric centre of expression appears ventral to the migrating eye (red dotted circle in N, O, R, T, U, W and X). The asymmetric *thrb* expression centre is located in the mesenchyme tissue that underlies the ventral blind side quadrant of the skull and extends the entire length of the eye to the distal mesenchyme tissue underlying the lower jaw (red dotted circle in N, O, R, T, U, W and X). Asymmetric expression of *thrb* increases up until climax (S3, G, H and T-V) and after metamorphosis (S4, I, J and W-Y) it is almost lost from both asymmetric and symmetric tissue. Notably, the *thrb* expression field in symmetric and asymmetric expressing regions coincides with that of *dio2* expression. Whole-mount lateral images of sole larvae head in pre- (S0, A and B), beginning (S1, C and D), early climax (S2, E and F) and the climax of metamorphosis (S3, G and H) and in post-metamorphic juveniles (S4, I and J). Vibratome sections of *thrb* WISH analysed larvae from pre- (S0, K-M), beginning (S1, N-O), early climax (S2, Q-S) and the climax of metamorphosis (S3, T-V) and in post-metamorphic juveniles (S4, W-Y). The cartoon of the head at the top of the images gives a representation of the position of the transverse head sections shown in the photographic plate. Vibratome sections correspond to the snout (K, N, Q, T and W), middle-eye field (L, O, R, U and X) and post-eye field (M, P, S, V and Y). In all vibratome sections cardinal points depict dorsal (D), ventral (V), ocular (O) and blind (B) sides. In all images scale bars represent 100µm.

Supplementary Figure 9. Blocking of sole metamorphosis by MMI decreases dio3 expression but not *mct8*. WISH expression analysis of dio3 in control (A and B) and MMI-treated larvae (C and D). Expression of dio3 in control is mostly found in skin (I-J) which is totally lost in MMI (L-N). In contrast, *mct8* expression in control (E and F) and MMI-larvae (G and H) is not changed. Vibratome sections of *mct8* WISH analysed samples further confirm that *mct8* expression is unchanged between control (O-Q) and MMI-treated (R-T) animals. Images A-H is of WISH analysed larvae (n=10/treatment/gene). Images I-T are of vibratome sections (n=3/treatment/gene) of WISH analysed larvae. In all vibratome sections cardinal points depict dorsal (D), ventral (V), ocular (O) and blind (B) sides. In all images the scale bars represent 100μm.

Supplementary Figure 10. Blocking of sole metamorphosis by MMI does not affect the expression of *thraa* and *thrab*. Images A-H are of WISH analysed larvae (n=10/treatment/gene). Images I-T are of vibratome sections (n=3/treatment/gene) of WISH analysed larvae. In all vibratome sections cardinal points depict dorsal (D), ventral (V), ocular (O) and blind (B) sides. In all images the scale bars represent 100μm.

Supplementary Figure 11. A TH-dependent asymmetric TH responsive centre drives asymmetric development of sole head. WISH analysis (n=10/group) and derived transverse sections (n=3/group) in normal climax metamorphosing sole larvae for *dio2* (A, B, I-K) and *thrb* (A, F, O-Q) shows the existence of a blind side-specific sub-optical expression field (red dotted circle). However, in MMI-blocked larvae *dio2* (C, D, L-N) and *thrb* (G, H, R-T) asymmetric expression is lost. Notably, MMI is associated with an increase in *dio2* symmetric expression fields but it is lost in the asymmetric centre highlighting two different mechanism occur for *dio2* expression in the sole head during development. In contrast *thrb* expression decreases in both symmetric and asymmetric expression fields thus highlighting that canonical regulation of *thrb* expression

by THs is in place. All scale bars represent 100μm. Cardinal orientation denotes dorsal (D), ventral (V), ocular (O) and blind (B) sides of the larvae.

Supplementary Figure 12. WISH negative controls (hybridized with sense probe) for *dio2*, *dio3*, *thraa*, *thrab*, *thrb*, *mct8* and MGP of sole larvae undergoing metamorphosis. Images presented correspond to the ocular side of larvae in post-metamorphic juveniles (S4). In all images the scale bars represent 100μm.

Supplementary Figure 13. qPCR expression analysis (n=5/gene/condition) of *dio2* (A), *dio3* (B), *thraa* (C), *thrab* (D), *thrb* (E) and *mct8* (F) in control (S3) and MMI-blocked metamorphosing sole larvae heads. Expression of *dio2*, *thraa*, *thrab* and *mct8* ($p>0.05$) does not change in the whole head of both control and MMI-blocked metamorphosing sole larvae heads. Inversely *dio3* ($p=0.0004$) and *thrb* ($p=0.0026$) expression is significantly decreased in sole larvae heads when metamorphosis is blocked by MMI.

Supplementary Figure 14. Validation of the polyclonal teleostean OSN (OSN) antisera by western blot. Purified recombinant His-tagged sea bream OSN (His-saOSN, 0.5 μg) was fractionated by SDS-PAGE (12%) in duplicate. One gel was used for Commassie-blue staining (A) and another for WB and blotted onto a PVDF membrane and immuno-stained (B) with: 1) saOSN anti-serum (1/50,000); 2) pre-absorbed saOSN anti-serum (1/50,000); and 3) pre-immune serum (1/50,000). An immunoreactive band of the expected size (≈ 37 kDa) for recombinant His-tagged saOSN was detected with the anti-saOSN sera and the signal was completely ablated when pre-absorbed saOSN anti-serum and pre-immune serum were used. The molecular weight marker (M) is indicated in kDa.

Movie 1. Animation of confocal microscopy generated volume reconstruction of control (S3-climax of metamorphosis) sole larvae head showing bone (green; OSN labeling) and muscle (magenta; myosin heavy chain labeling) tissues. The developing asymmetric pseudomesial (pdm) bone is shown in pink to highlight

its asymmetric development. No asymmetric muscle development is observed. The scale bar represents 500μm. D- dorsal side; O-ocular side; B-blind side; prm-paramesial bone; eth-ethmoid; pdm-pseudomesial bone. See Fig.1 for details.

Movie 2. Animation of confocal microscopy generated volume reconstruction of MMI-treated (metamorphosis blocked) sole larvae head showing bone (green; OSN labeling) and muscle (magenta; myosin heavy chain labeling) tissues. In contrast to control larvae (Movie 1) the pseudomesial bone does not develop (pink asterisks denote head region where pdm should be developing and the red asterisks denotes the corresponding region on the ocular side). No asymmetric development occurs. The scale bar represents 500μm. D- dorsal side; O-ocular side; B-blind side; prm-paramesial bone; eth-ethmoid; pdm-pseudomesial bone. See Fig.1 for details.

Movie 3. Animation of confocal microscopy generated volume reconstruction of control (S3-climax of metamorphosis) sole larvae head showing nerve (acetylated β-tubulin) and synaptic vesicle (SV2) labeling. The olfactory epithelia and lobe and the eyes are the only nervous tissue structures that become asymmetric during metamorphosis and this occurs in exactly the same region where asymmetric ossification occurs (Movie 1; Fig. 1). The scale bar denotes 500μm. D- dorsal side; O-ocular side; B-blind side. See Fig.2 for details.

Movie 4. Animation of confocal microscopy generated volume reconstruction of MMI-treated (metamorphosis blocked) sole larvae head showing nerve (acetylated β-tubulin) and synaptic vesicle (SV2) labeling. Concomitant with the lack of development of the pdm (Movie 1 and Fig. 1) no asymmetric development of the olfactory epithelia, bulb and eyes is observed on metamorphosed blocked larvae. The scale bar denotes 500μm. D- dorsal side; O-ocular side; B-blind side. See Fig.2 for details.

Supplementary Figure 1

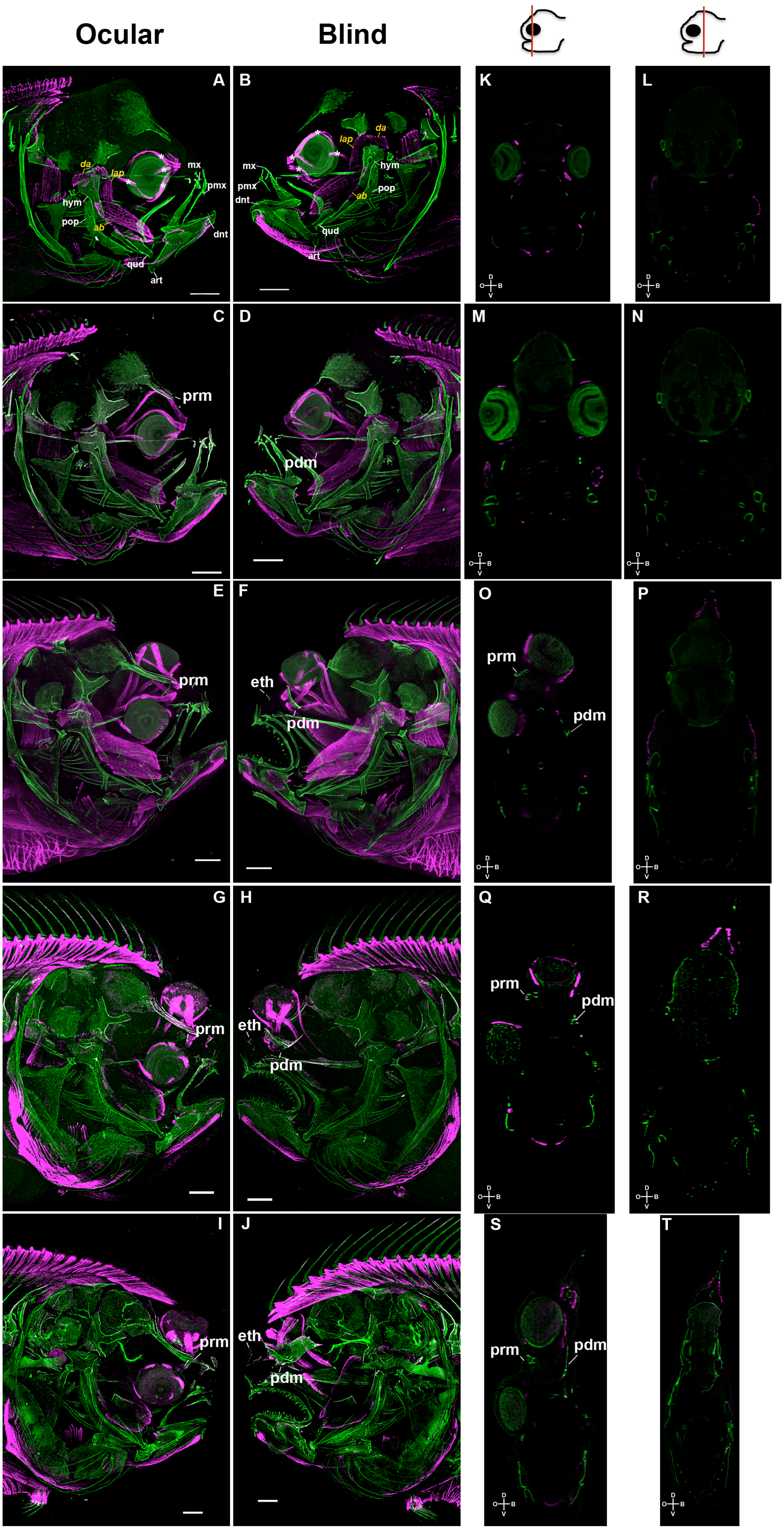

Supplementary Figure 2

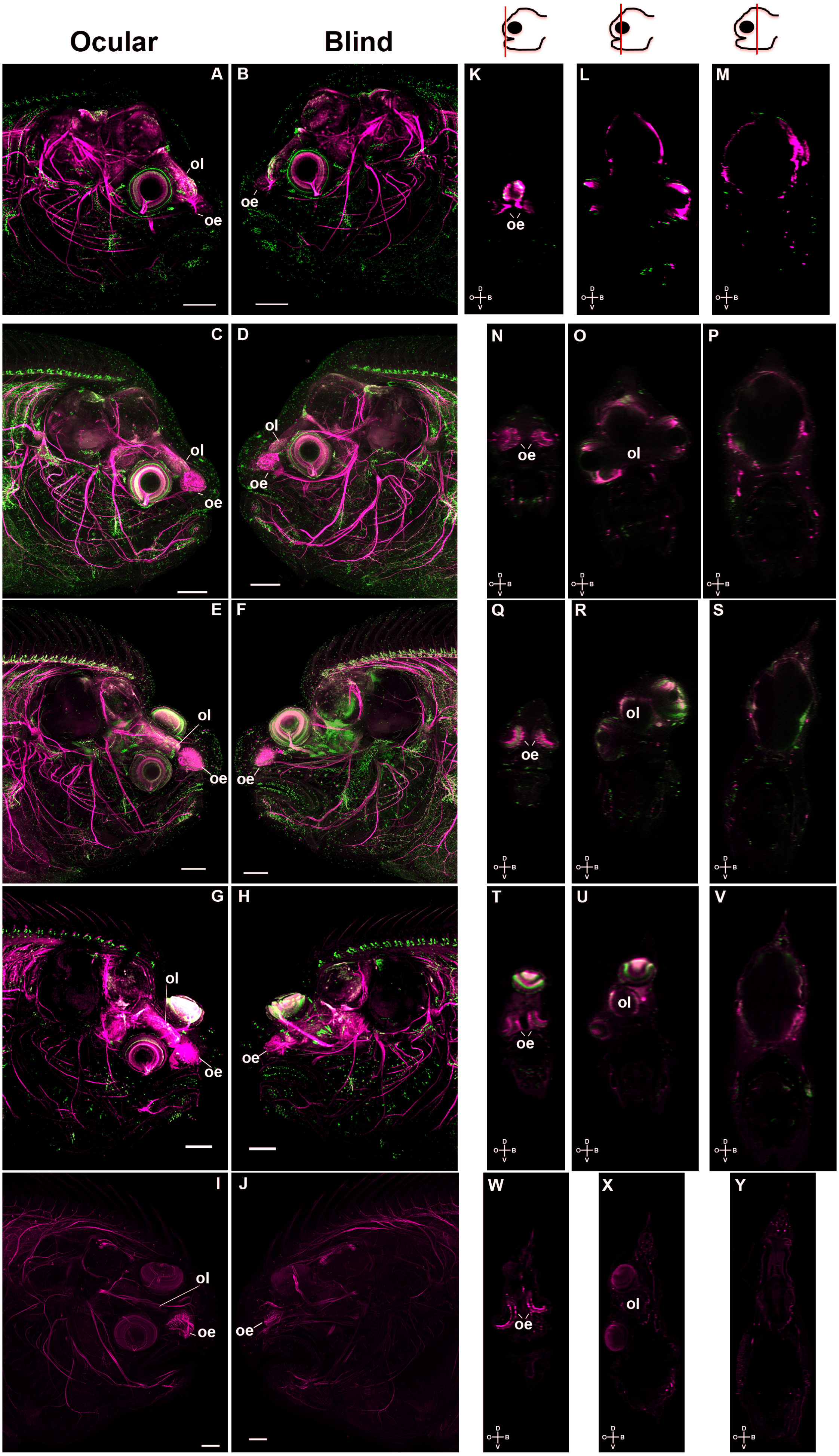

Supplementary Figure 3

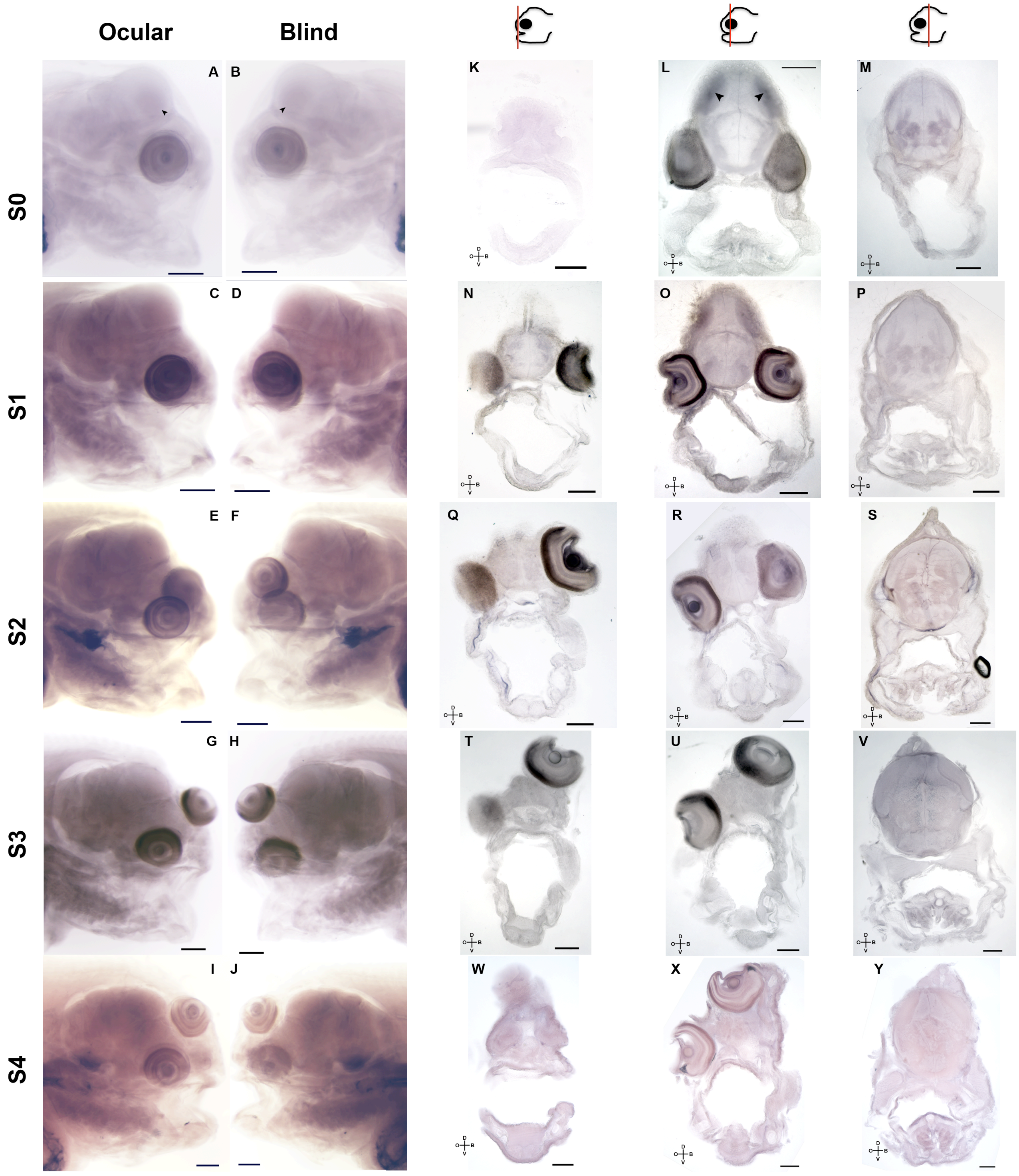

Supplementary Figure 4

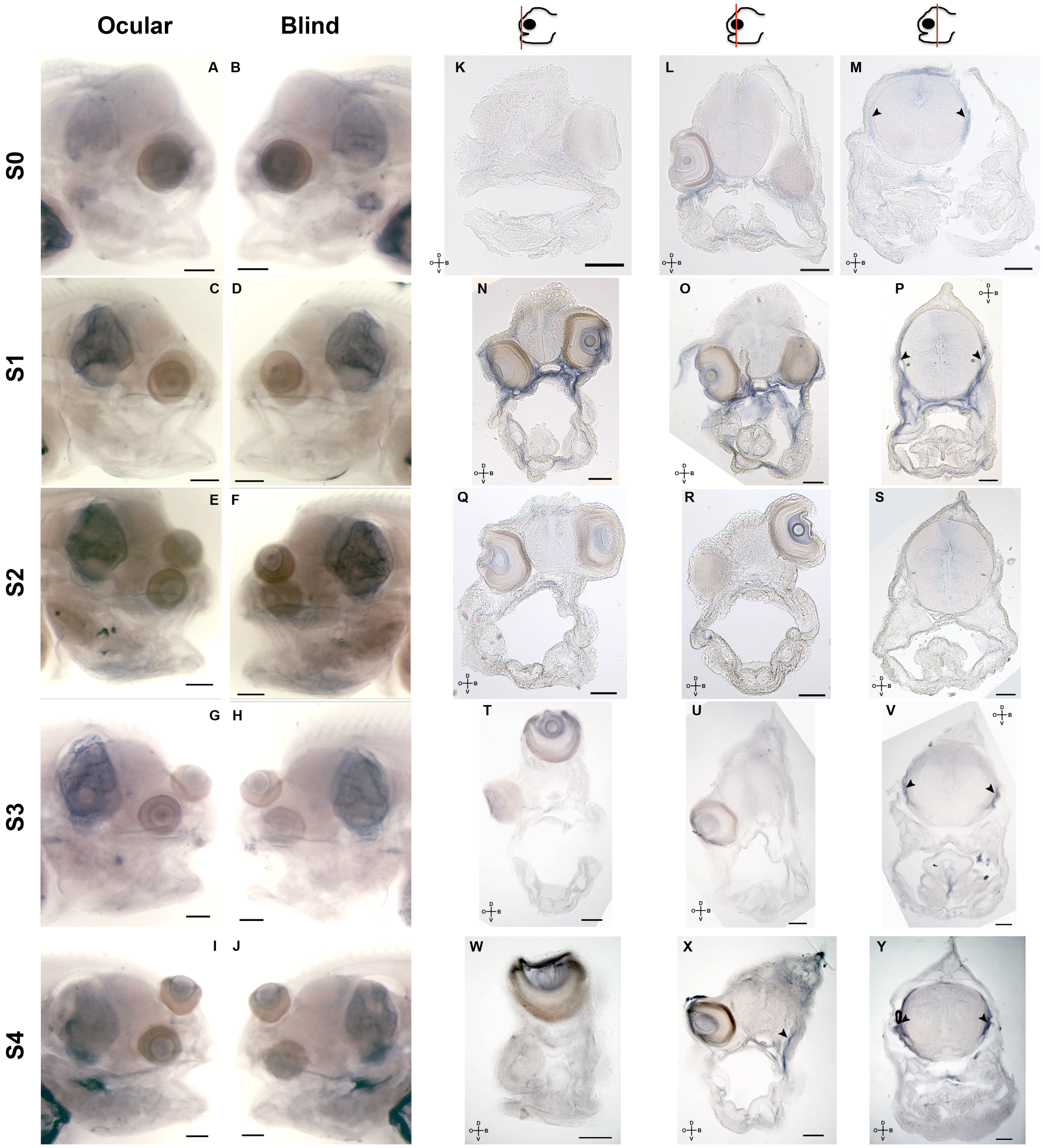

Supplementary Figure 5

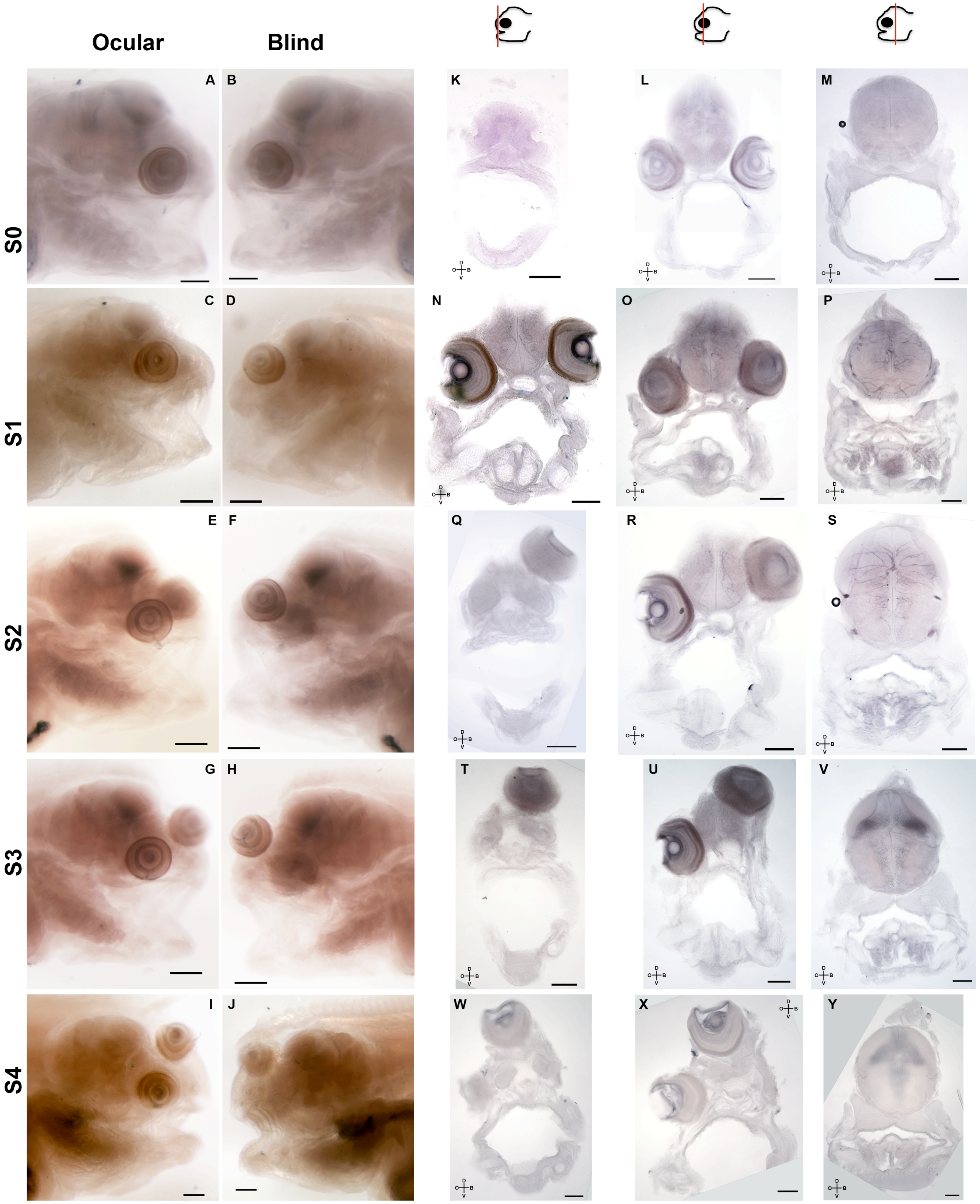

Supplementary Figure 6

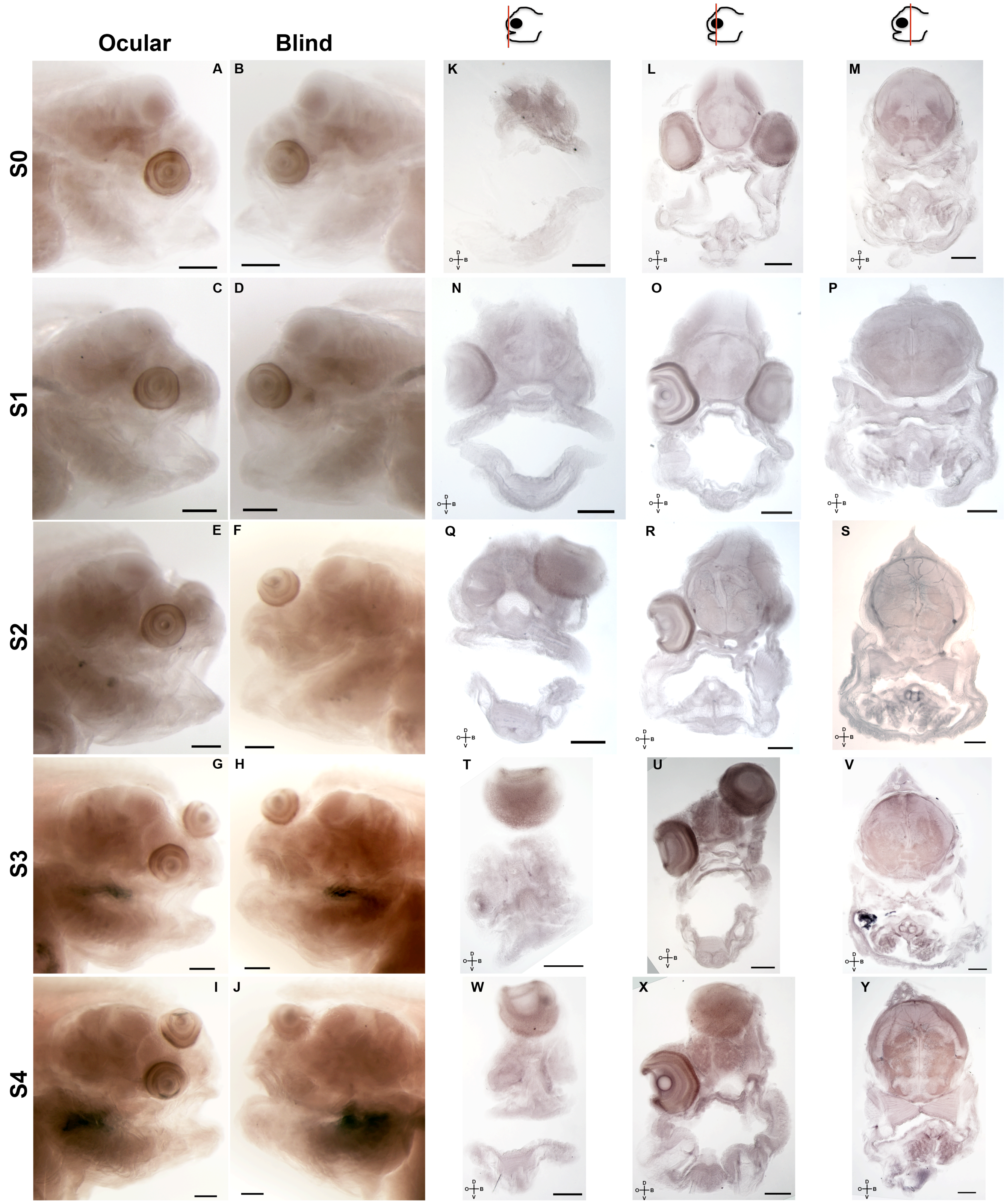

Supplementary Figure 7

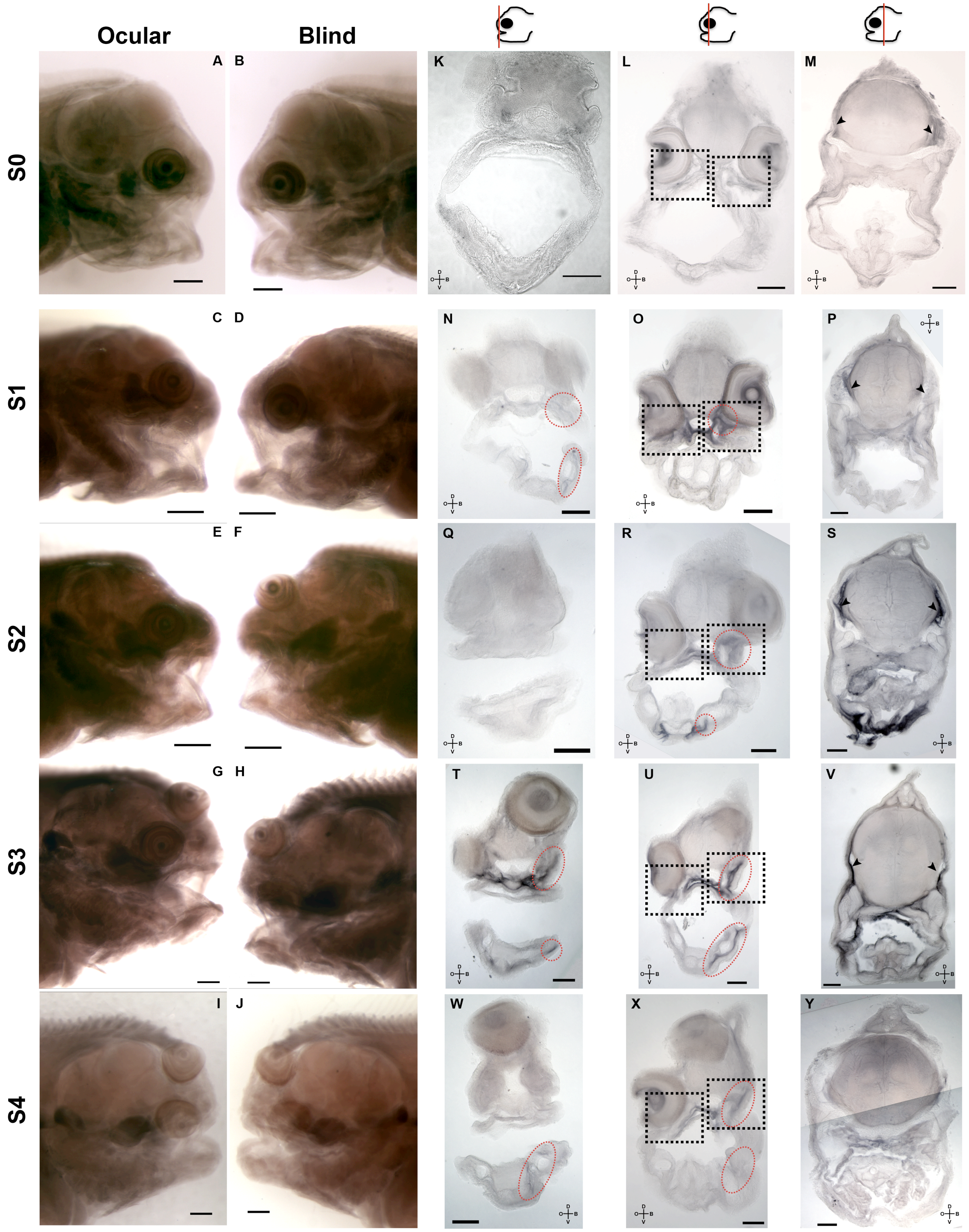

Supplementary Figure 8

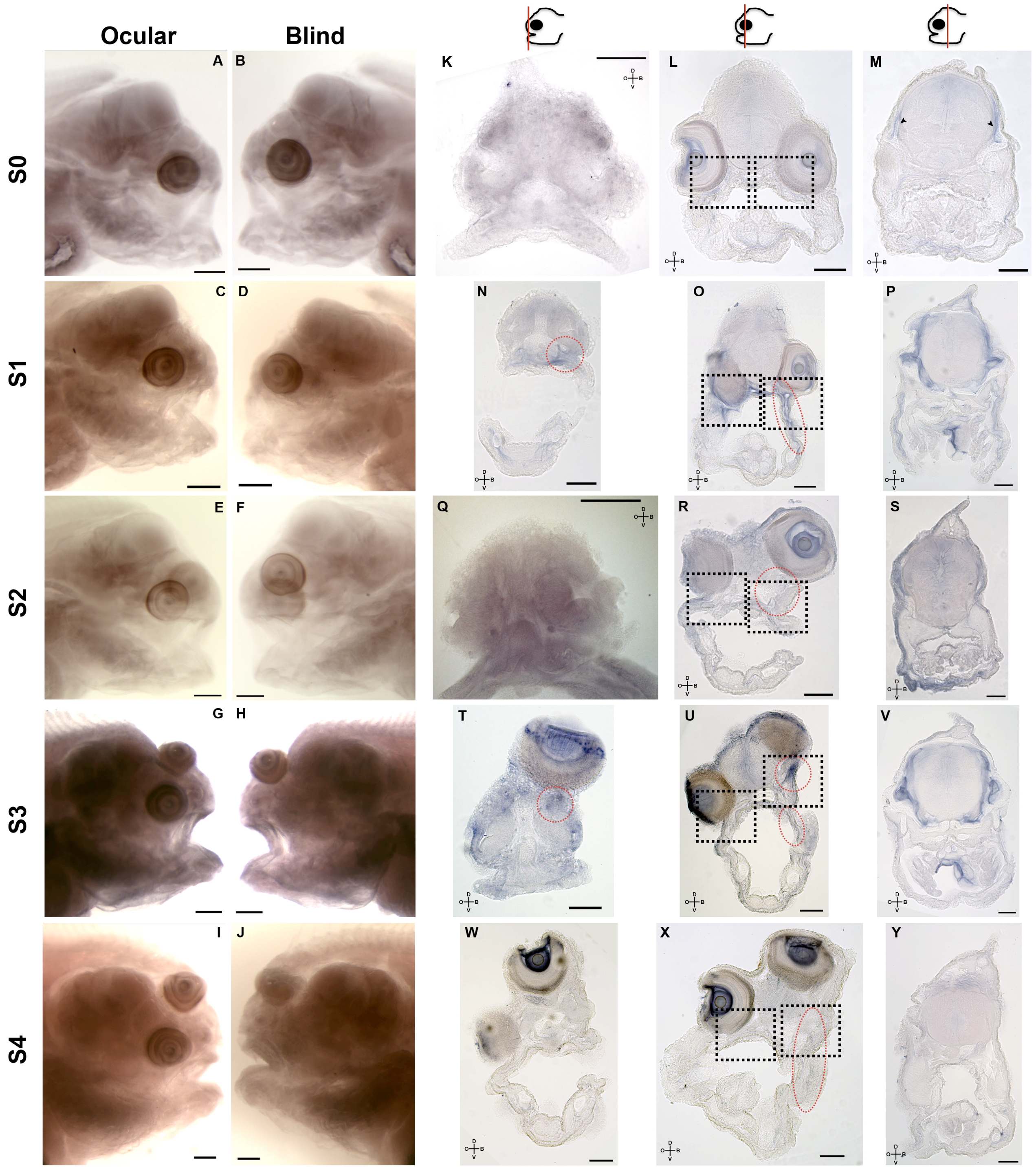

Supplementary Figure 9

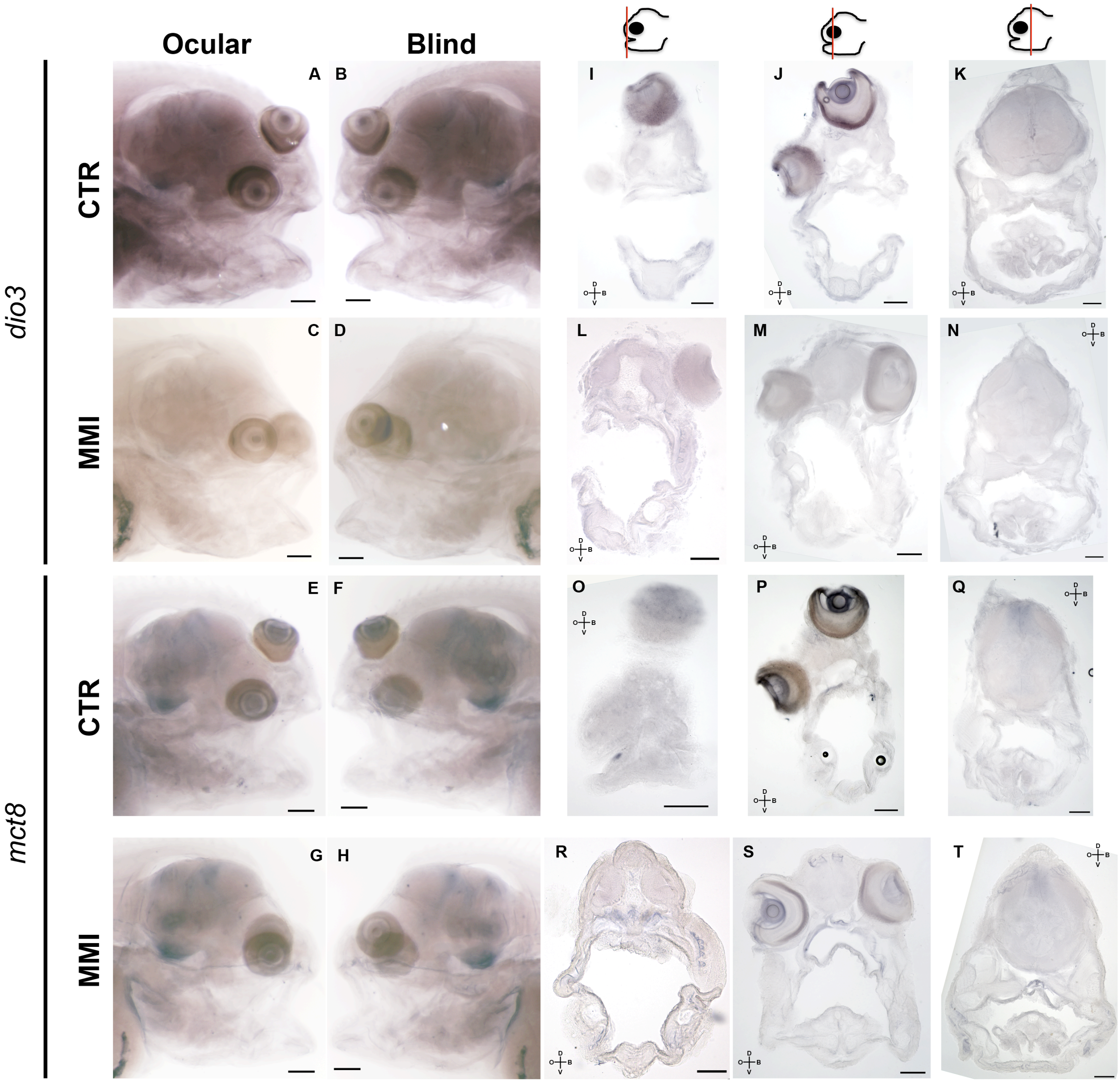

Supplementary Figure 10

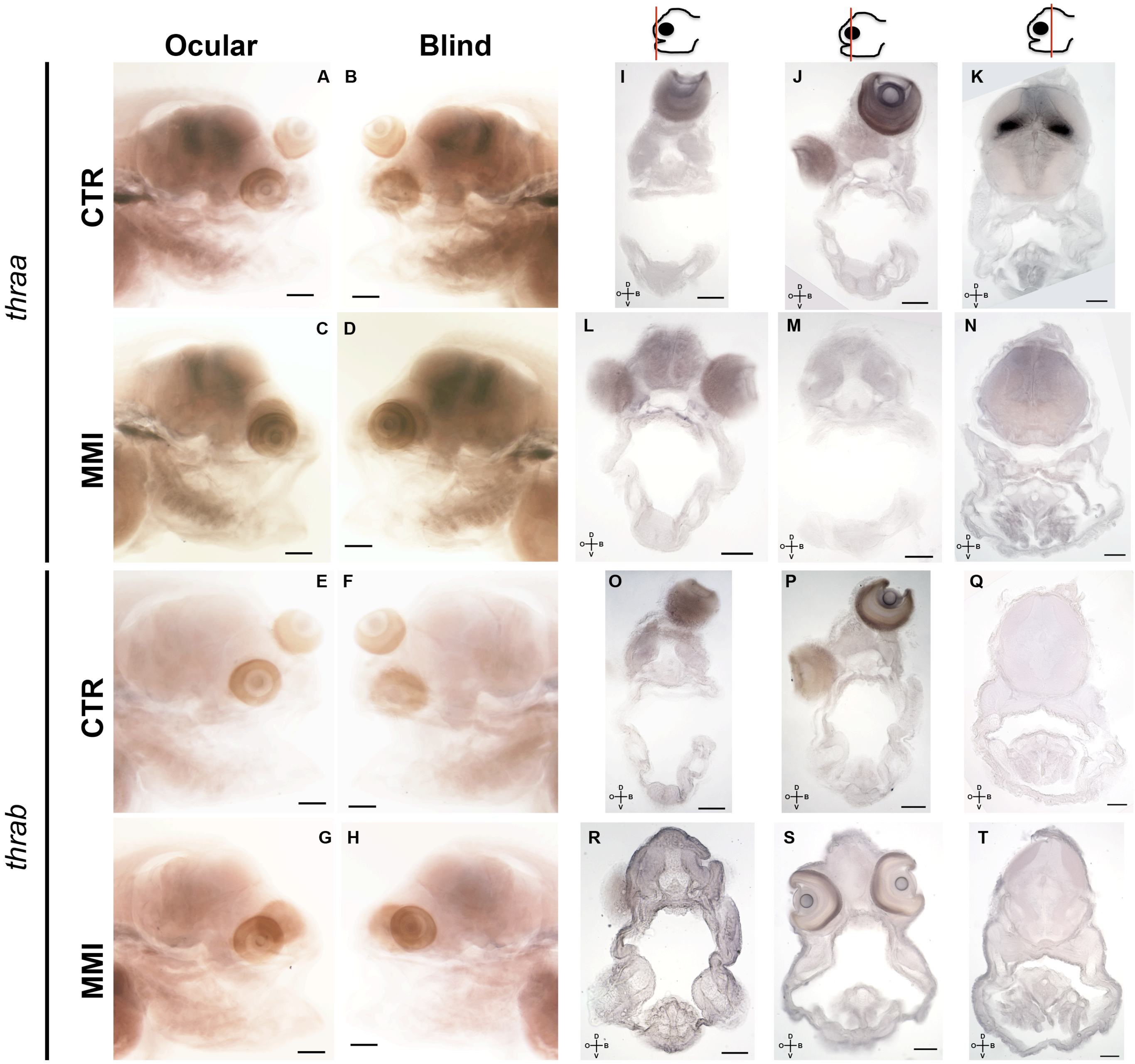

Supplementary Figure 11

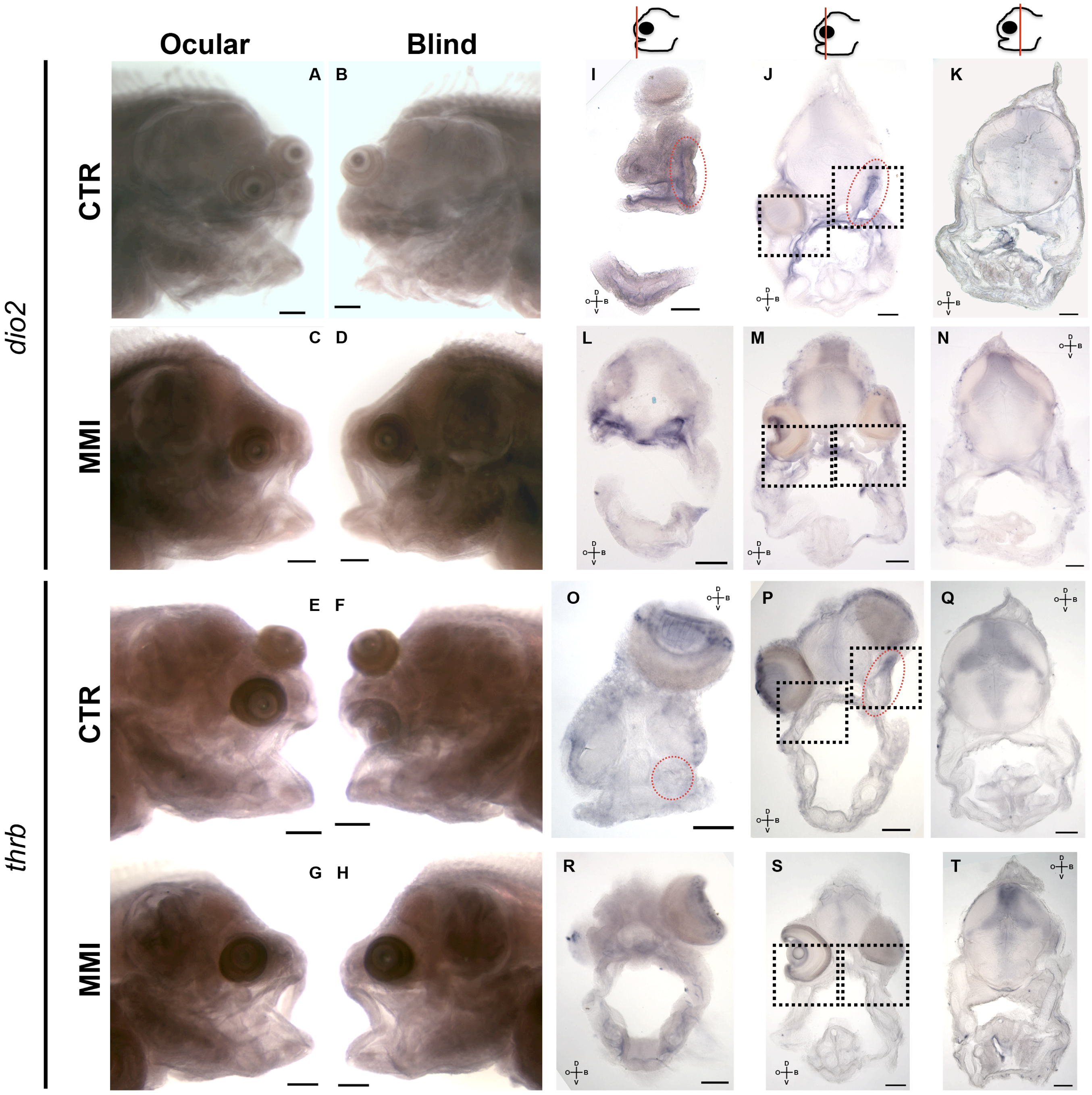

Supplementary Figure 12

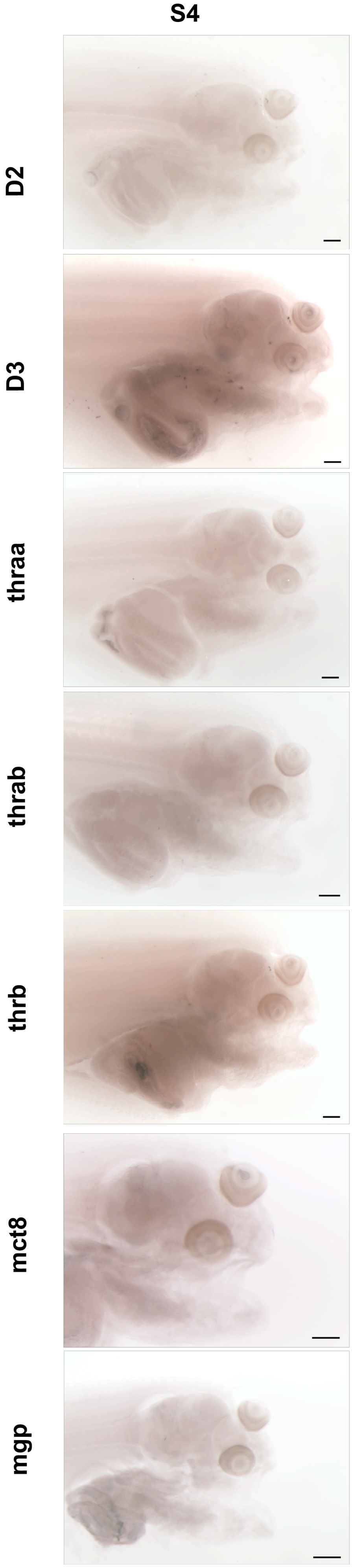

Supplementary Figure 13

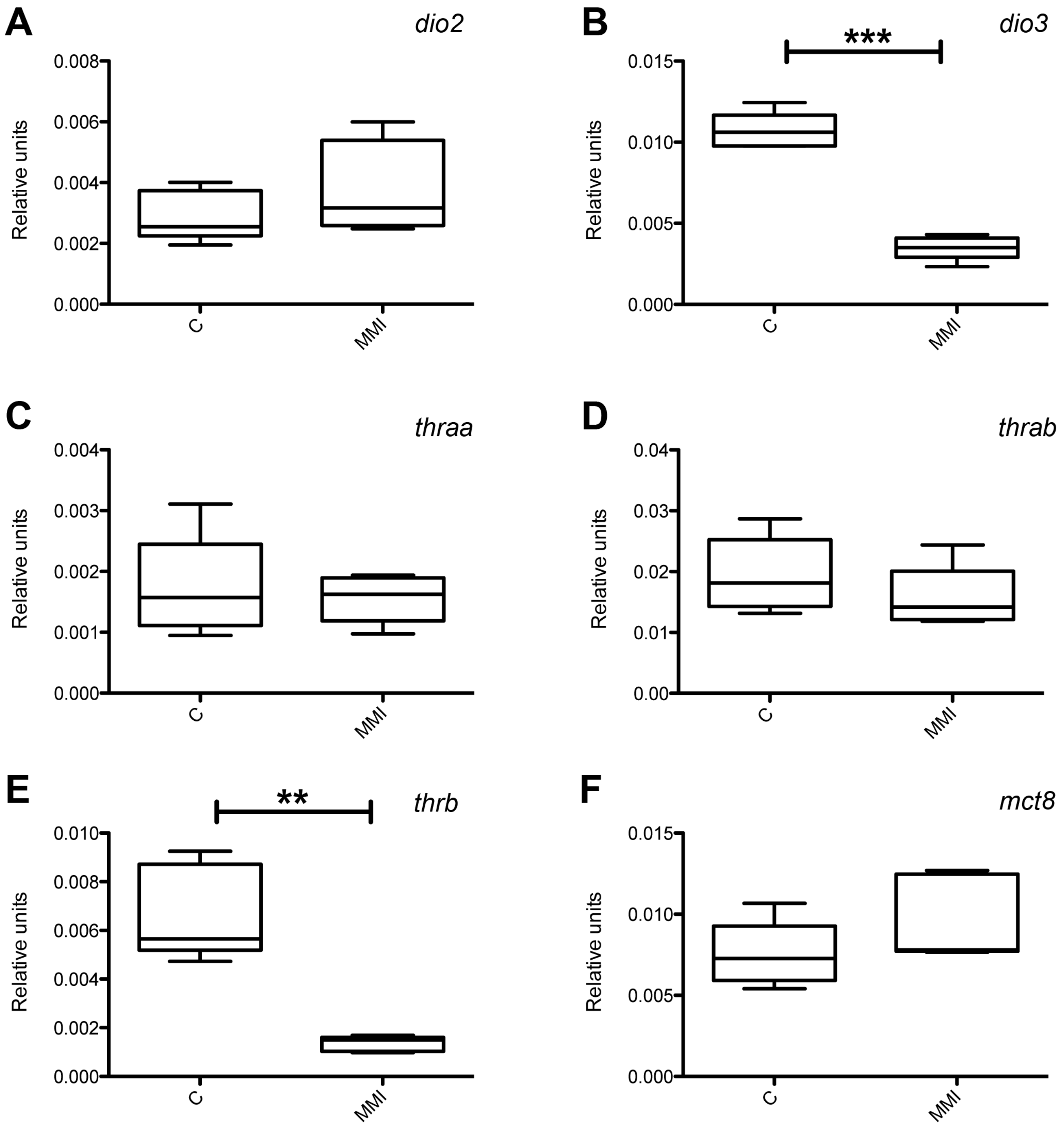

Supplementary Figure 14

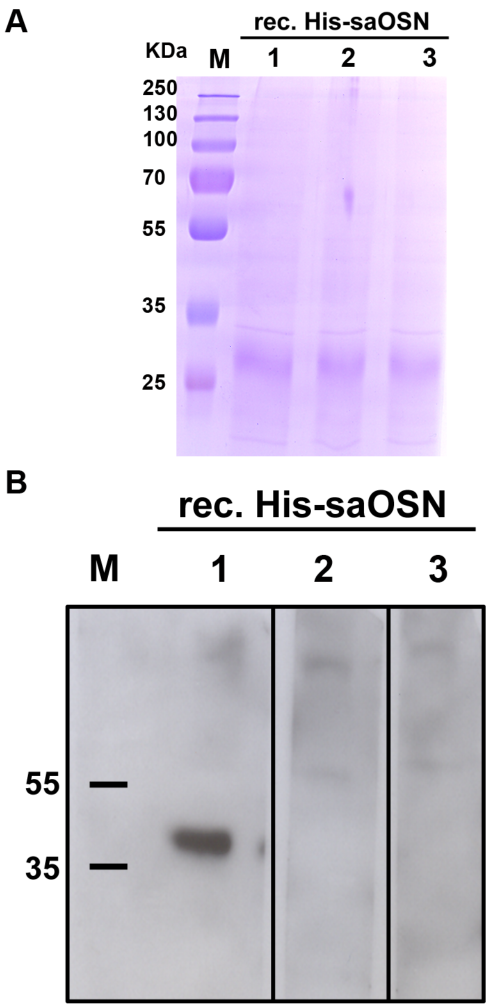

Supplementary table 1. qPCR primer sequences for sole 18s, D2, D3, thraa, thrab thrb and MCT8

| Gene | Forward primer (5'-3') | Reverse primer (5'-3') |
|---|---|---|
| 18s | AACCAGACAAATCGCTCCAC | CCTGCGGCTTAATTTGACTC |
| D2 | TGAACTCAGGCCAAAACTGC | CGGCATAAATCCAGGTGAAG |
| D3 | TGGCTGGAGCAATACAAGAG | TGGACACTTGAAGCAGAACG |
| thraa | TTCCCGTCTTCTCTTCATGC | AGCGAAAGGTCACAAACAGG |
| thrab | CAGCGAGTTCACCAAGATCA | CTCACTCTCGGGATCGTAGC |
| thrb | AGAAGGGCCATCACATGAAC | ACCCGCACTGTAAAGGAATG |
| MCT8 | CTTGGCTATGGGCATGATCT | GATCCGCAGCCAAACAGTAT |

Supplementary table 2. Cloning primers sequences for sole D2, D3, thraa, thrab, thrb, MCT8 and MGP

| Gene | Forward primer (5'-3') | Reverse primer (5'-3') |
|---|---|---|
| D2 | TGCGGTGCATCTGGAACAGCTTCC | GTTGTTGTTGTCGTCGTCGTCG |
| D3 | CCAGTCGCATCCTATCTACAGG | CACACACACACACGCACACACTG |
| thraa | GAGGCAGGAGTATAGCAACAG | CTGGTCTTCACAAGGCAGCTCTG |
| thrab | GCCTCAGTCTCTCATAATTGGCTGC | GAGCGGAGGGAAGAGTTCGTTGG |
| thrb | TAGTTGTACCCAGACGTCTGAGG | CCATCGGTCAGTCCTCGAAGAC |
| MCT8 | CGTCTGCTGGTGGAAGTTGAC | TCATGGTGAGAACGCAGCCGT |
| MGP | CCAGGATGAGGAGCCTTCTTC | GACAGTTTAGTATCGACGGATC |
